# Mitochondrial function provides instructive signals for activation-induced B-cell fates

**DOI:** 10.1038/ncomms7750

**Published:** 2015-04-10

**Authors:** Kyoung-Jin Jang, Hiroto Mano, Koji Aoki, Tatsunari Hayashi, Akihiko Muto, Yukiko Nambu, Katsu Takahashi, Katsuhiko Itoh, Shigeru Taketani, Stephen L. Nutt, Kazuhiko Igarashi, Akira Shimizu, Manabu Sugai

**Affiliations:** 1 Department of Experimental Therapeutics, Institute for Advancement of Clinical and Translational Science, Kyoto University Hospital, 54 Shogoin-Kawahara-cho, Sakyo-ku, Kyoto 606-8507, Japan; 2 Division of Pharmacology, School of Medicine, University of Fukui, 23-3 Matsuokashimoaizuki, Eiheiji-cho, Yoshida-gun, Fukui 910-1193, Japan; 3 Department of Biochemistry, Tohoku University Graduate School of Medicine, Seiryo-machi 2-1, Aoba-ku, Sendai, Miyagi 980-8575, Japan; 4 CREST, Japan Science and Technology Agency, Seiryo-machi 2-1, Aoba-ku, Sendai, Miyagi 980-8575, Japan; 5 Department of Oral and Maxillofacial Surgery, Kyoto University Hospital, 54 Shogoin-Kawahara-cho, Sakyo-ku, Kyoto 606-8507, Japan; 6 Department of Clinical Molecular Biology, Graduate School of Medicine, Kyoto University, 54 Shogoin-Kawahara-cho, Sakyo-ku, Kyoto 606-8507, Japan; 7 Department of Biotechnology, Kyoto Institute of Technology, Matsugasaki, Sakyo-ku, Kyoto 606-8585, Japan; 8 The Walter and Eliza Hall Institute of Medical Research, Melbourne, Victoria 3052, Australia; 9 Department of Medical Biology, University of Melbourne, Victoria 3010, Australia; 10 Division of Molecular Genetics, Department of Biochemistry and Bioinformative Sciences, School of Medicine, University of Fukui, 23-3 Matsuokashimoaizuki, Eiheiji-cho, Yoshida-gun, Fukui 910-1193, Japan

## Abstract

During immune reactions, functionally distinct B-cell subsets are generated by stochastic processes, including class-switch recombination (CSR) and plasma cell differentiation (PCD). In this study, we show a strong association between individual B-cell fates and mitochondrial functions. CSR occurs specifically in activated B cells with increased mitochondrial mass and membrane potential, which augment mitochondrial reactive oxygen species (mROS), whereas PCD occurs in cells with decreased mitochondrial mass and potential. These events are consequences of initial slight changes in mROS in mitochondria^high^ B-cell populations. In CSR-committed cells, mROS attenuates haeme synthesis by inhibiting ferrous ion addition to protoporphyrin IX, thereby maintaining Bach2 function. Reduced mROS then promotes PCD by increasing haeme synthesis. In PCD-committed cells, Blimp1 reduces mitochondrial mass, thereby reducing mROS levels. Identifying mROS as a haeme synthesis regulator increases the understanding of mechanisms regulating haeme homeostasis and cell fate determination after B-cell activation.

## Abstract (web summary)

Cell fate choices are often based on amplification of noise. Here the authors show that small initial differences in mitochondrial reactive oxygen species lead to bigger changes in mitochondrial mass and membrane potential, which then determine plasma cell fate choice of activated B cells.

## Graphical abstract

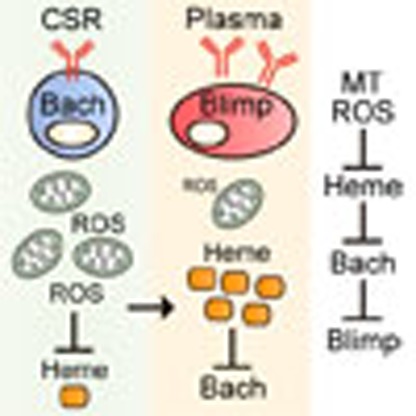

On antigen challenge, naïve B lymphocytes undergo diversification of their antigen receptor via somatic hypermutation (SHM), alteration of immunoglobulin function by class-switch recombination (CSR)^1^,^2^,^3^,^4^,^5^,^6^,^7^ and differentiation into antibody-secreting plasma cells or memory B cells^8^,^9^,^10^,^11^,^12^,^13^. Although several important transcription factors involved in these processes have been identified, the interrelations in the regulatory network that determine cell fates after B-cell activation remain elusive^14^,^15^,^16^,^17^. Pax5 and Bach2 are required for CSR because ablations of these genes in B cells destroy the ability of the cell to undergo CSR^2^,^18^. Pax5 and Bach2 also inhibit plasma cell differentiation (PCD) by inhibiting the transcription of *Blimp1*, a master gene for PCD^2^,^19^,^20^,^21^,^22^. Irf4 and Xbp1 act in concert with Blimp1 to establish plasma cell fate^8^,^11^,^13^,^23^,^24^. However, Blimp1 represses Pax5 expression in differentiating plasma cells^9^,^10^. The transcription factor Irf4 plays a pivotal regulatory role in these processes because it is required for affinity maturation, CSR and PCD^11^,^12^,^13^. An intermediate amount of Irf4 induces the expression of Bcl6 and activation-induced cytidine deaminase (AID), whereas a high amount induces the expression of Blimp1 (refs 12, 13). CSR and SHM are initiated by the AID-dependent cytidine deamination of DNA in immunoglobulin loci^1^,^3^,^4^,^5^,^6^. Bcl6 is required for germinal-centre (GC) B-cell development. During the GC reaction, B cells undergo SHM at their immunoglobulin loci, and high-affinity immunoglobulin-bearing B cells are selected in a process called affinity maturation^25^,^26^,^27^,^28^,^29^. Mutually exclusive expression of Bcl6 and Blimp1 is established by the mutual inhibition of expression. Although the essential gene sets for various B-cell fates after activation and a regulatory network that inhibits unselected fates have been described, the instructive signals for the initiation of the respective B-cell fates or transitions between them are not completely known.

Metabolism encompasses the generation of energy via catabolism and of macromolecules via anabolism. Recent studies have indicated more complex roles of metabolic pathways or metabolites in cell differentiation and function^30^,^31^,^32^,^33^. A shift towards glycolysis occurs in macrophages activated by lipopolysaccharide (LPS), inducing interleukin-1β production in a hypoxia-inducible factor 1-alpha-dependent manner. Inhibition of glycolysis using 2-deoxyglucose suppresses interleukin-1β production via the reduction of succinate, an intermediate of the tricarboxylic acid (TCA) cycle or the γ-aminobutyric acid shunt, and stabilizes HIF-1α (ref. 31). A strong bias towards glycolysis over mitochondrial metabolism has also been observed in haematopoietic stem cell (HSC) maintenance^34^, Th17 differentiation, tumour cell functions and interferon-γ production in activated T cells^30^. However, regulatory T cells display a mixed metabolism that includes lipid oxidation, oxidative phosphorylation and glycolysis^30^. In addition to generating various metabolites in mitochondria, mitochondria-derived reactive oxygen species (mROS) are now understood to function as signalling molecules^35^. This indicates that mitochondria play fundamental roles in cell differentiation and function. In activated B cells, haeme strongly induces PCD by inhibiting Bach2 function^36^.

Because activated B cells differentiate into various cell types in a stochastic manner^15^, we speculated that stochastic changes in some metabolic pathways would provide instructive signals to activated B cells. In this study, we investigated the functions of mitochondria in fate determination of activated B cells and attempted to identify the signals directing B-cell fate towards CSR or PCD. We first observed that differences in mitochondrial status predicted the direction of the committed cells toward either pathway. CSR occurred in activated B cells with increased mitochondrial mass and membrane potential, whereas PCD occurred in cells with decreased mitochondrial mass and potential. However, differences in mitochondrial mass and membrane potential were the consequences of slight initial changes in generating mROS within the mitochondria^high^ B-cell population. Activated mitochondria^high^ B cells with higher amounts of mROS maintained increased mitochondrial mass and membrane potential, whereas cells with lower amounts displayed decreased mitochondrial mass and potential. Thus, slight differences in mROS were the initial signal in cell fate determination after B-cell activation. Notably, the choice for the B-cell fate of PCD or CSR was dependent on mitochondrial functional activity that regulated an intracellular ROS level, which suppressed haeme synthesis. These results suggest that mitochondria contribute to B-cell fate determination via the integration of instructive and cell-intrinsic stochastic signals by regulating haeme and ROS levels.

## Results

### Activation-dependent generation of three B-cell populations

**Figure 1:**
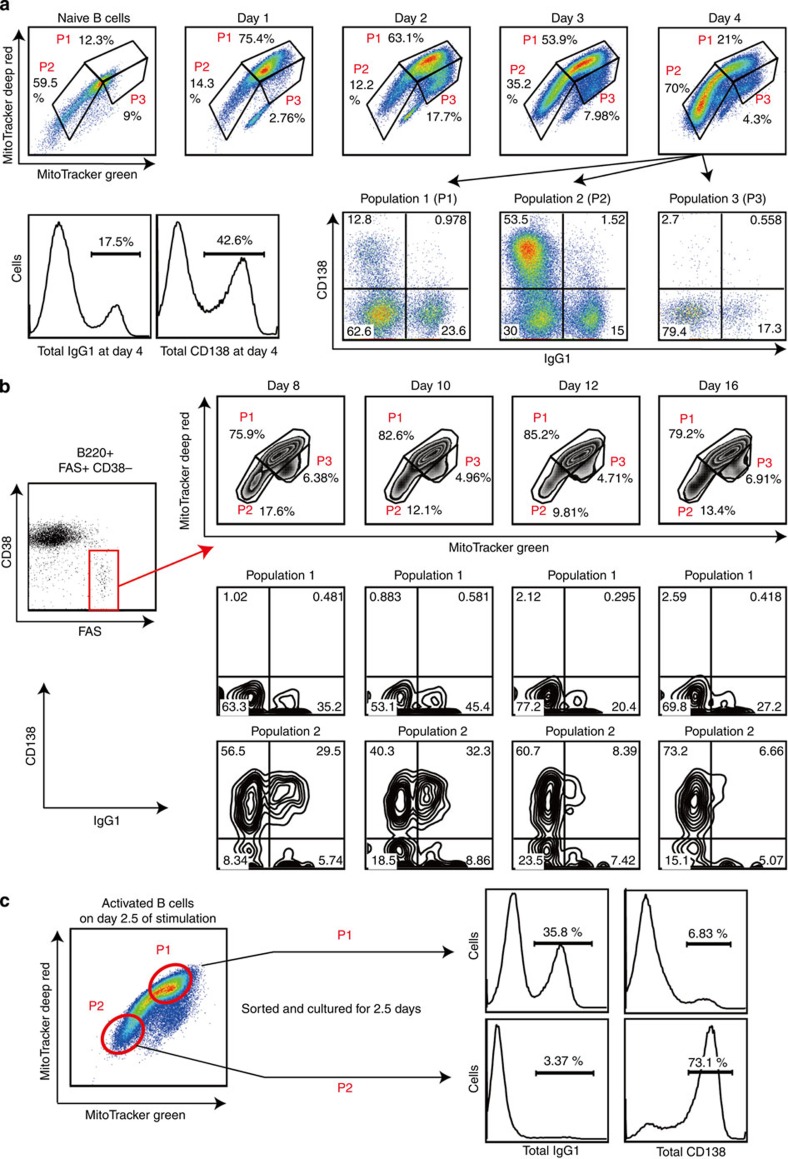
Activated B cells are subdivided into three groups according to the mitochondrial status. (**a**) Flow cytometric analysis of mitochondrial membrane potential and size monitored by MitoTracker staining on the indicated day (top) or differentiation of the B cells monitored by CD138 and IgG1 expression on day 4 (bottom) in LPS+IL-4-stimulated B cells. (**b**) Flow cytometric analysis of the mitochondrial status on the indicated day after immunization (top) with NP-CGG and the differentiation status of population 1 (middle) and population 2 (bottom) in GC B cells (B220+CD38−FAS+). (**c**) Diagrammatic representation of experimental overview. Flow cytometric analysis of differentiation of sorted P1 and P2 cells. Data shown are representative of three independent experiments.

Because alterations of metabolic pathways or metabolites are involved in lineage commitment and cell differentiation^31^,^32^,^33^, we hypothesized that intracellular metabolism plays some role in the fate determination of the activated B cells. To test this possibility, we investigated the involvement of mitochondria in B-cell fate determination, considering that mitochondria produce various metabolites including ATP, haeme and iron–sulfur clusters. First, we evaluated total mitochondrial mass and membrane potential after B-cell activation *in vitro*^37^ using MitoTracker Green and DeepRed dyes, respectively. Mitochondrial mass and membrane potential were increased, and a relatively uniform population (called population 1, P1) was generated on day 1 of stimulation with LPS+IL-4 or anti-CD40+IL-4 (Fig. 1a and Supplementary Fig. 1b). On day 3 of stimulation, cells showing intermediate levels of mitochondrial mass and membrane potential (population 2, P2) increased in frequency. To investigate the relationship between mitochondrial status and cell fates, we examined the mitochondrial status of class switched cells (IgG1^+^ cells) and plasma cells (CD138^+^ cells) on day 4 of stimulation. IgG1^+^ cells preferentially belonged to the P1 population and CD138^+^ cells to the P2 population (Fig. 1a and Supplementary Fig. 1a–c), suggesting an association between P1 cells and CSR and between P2 cells and PCD. Another minor population, called P3 cells, also expressed IgG1. When the same assays were performed using TMRM dye, instead of MitoTracker DeepRed, essentially the same results were obtained (Supplementary Fig. 2a). Next we evaluated total mitochondrial mass and membrane potential after B-cell activation *in vivo*. Again, we found P1, P2 and P3 cell populations among GC B cells during the T-cell-dependent immune reaction. P1 cells preferentially expressed IgG1, whereas P2 cells expressed CD138 (Fig. 1b). In this case, IgG1-expressing plasmablasts were also observed among P2 cells. This finding indicated that PCD occurred after IgG1 switching (Fig. 1b), because time-course analysis indicated that P2 cells were generated later than P1 cells after B-cell activation *in vitro* (Fig. 1a and Supplementary Fig. 1b). Same assays were performed using TMRM dye, instead of MitoTracker DeepRed, and essentially the same results were obtained (Supplementary Fig. 1d). CD138^+^ cells were also enriched in P2 populations within GL7^+^ GC B cells (Supplementary Fig. 3a). We further examined mitochondrial status of splenic plasma cells in the same mice as used for Fig. 1b. Proportions of P2 populations were increased in plasma cells (Supplementary Fig. 3b). In the T-cell-independent immune response, plasma cells were also observed among P2 cells, but IgG3-expressing cells were observed among P1 cells (Supplementary Fig. 3c). Thus, there was a strong association between mitochondrial status and B-cell fate determination. To evaluate this further, we investigated the differential abilities of differentiation of P1 and P2 cells towards CSR and PCD. To this end, we collected undifferentiated P1 and P2 cells (indicated populations in Fig. 1c) that did not express IgG1 and CD138 and stimulated them to differentiate. Consistent with the above results (Fig. 1a,b), IgG1 was expressed in more cells derived from P1 than from P2 cells (Fig. 1c), whereas CD138 was expressed in more cells derived from P2 than from P1 cells (Fig. 1c). These results suggested that undifferentiated cells found in P1 and P2 cell populations were committed to CSR and PCD, respectively.

### Modulation of mitochondrial function affects B-cell fate

**Figure 2:**
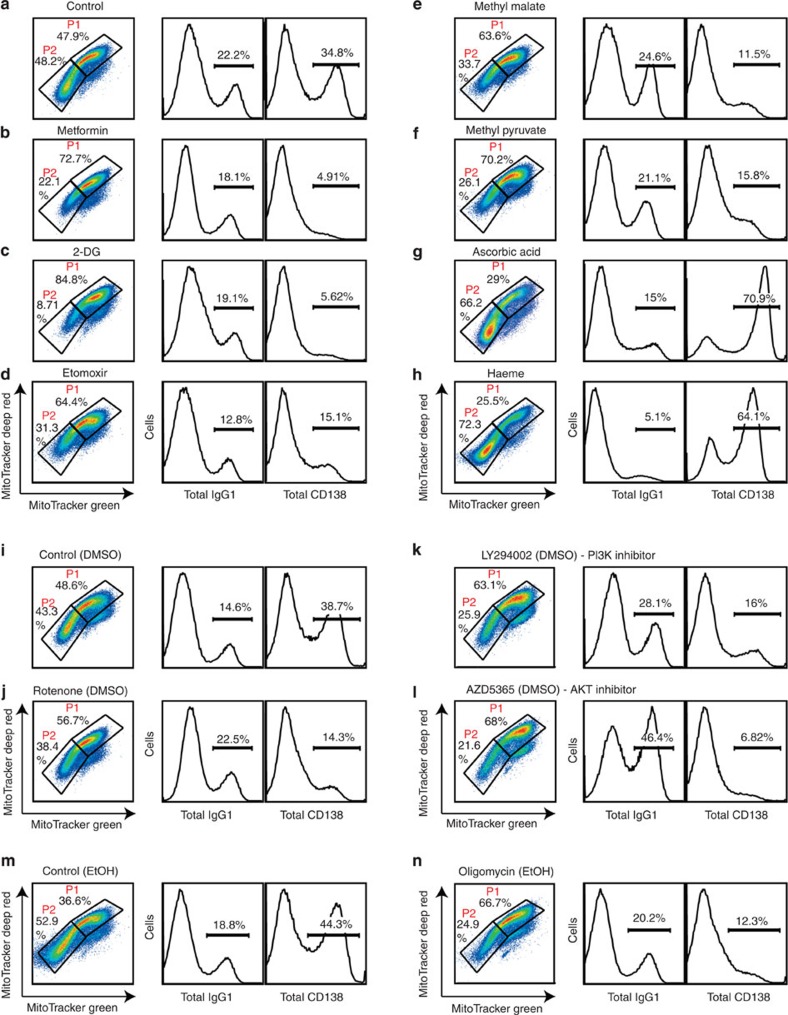
Association of mitochondrial status with activated B-cell fate. Flow cytometric analysis of mitochondrial status monitored by MitoTracker staining (left) or differentiation monitored by CD138 (right) and IgG1 (middle) expression after 4 days of culture with LPS+IL-4 in the presence or absence of the indicated reagents. **a** is the control for **b**–**h**. **i** is the control for **j**–**l**. **m** is the control for **n**. Data shown are representative of three independent experiments. DMSO, dimethylsulphoxide.

To investigate the contribution of mitochondrial metabolism to B-cell fate determination, we blocked key enzymes of the respiratory chain of mitochondria to reduce ATP levels. The number of cells in the P1 cell fraction was increased by the addition of the complex I inhibitors rotenone/metformin or the complex V inhibitor oligomycin, whereas PCD was strongly suppressed (Fig. 2a,b,i,j,m,n and Supplementary Fig. 4a). We also inhibited the major metabolic pathways in mitochondria to examine the involvement of distinctive catabolic pathways of glucose or fatty acids in activated B-cell fate determination. We found increases in P1 cell numbers and decreases in P2 cell numbers after treatment with 2-deoxyglucose, a glucose analogue that inhibits glycolysis, and etomoxir, an inhibitor of fatty acid oxidation (Fig. 2a,c,d and Supplementary Fig. 4a). Similarly, increased P1 cell numbers and decreased P2 cell numbers were observed after treatment with methyl pyruvate, which provides substrates for the TCA cycle, and methyl malate, which generates NADPH (Fig. 2a,e,f and Supplementary Fig. 4a). In contrast, P2 cell generation and PCD were enhanced by the addition of the antioxidant ascorbic acid, whereas CSR was suppressed (Fig. 2a,g and Supplementary Fig. 4a).

Treatment of activated B cells with inhibitors of the phosphatidylinositol 3-kinase (PI3K)–Akt pathway increased P1 cell numbers, enhanced CSR, reduced P2 cell numbers and suppressed PCD (Fig. 2i,k,l and Supplementary Fig. 4a). These results were consistent with earlier findings that the PI3K–Akt pathway inhibits CSR and promotes PCD^38^.

We further investigated the relationship between haeme and mitochondrial function in the control of B-cell fate determination because haeme is a strong inducer of PCD via the inhibition of Bach2 function^36^. Addition of haeme promoted skewed P2 cell generation (Fig. 2a,h and Supplementary Fig. 4a). Because CSR and PCD are influenced by cell proliferation, effects of various reagents on the number of cell divisions were assessed using carboxyfluorescein succinimidyl ester (CFSE)-labelled B cells. As shown in Supplementary Fig. 5a–d,f,h–k, the proliferation of cells was less affected by the presence of various reagents. Methyl pyruvate inhibited cell proliferation but affected only PCD (Supplementary Fig. 5g). For etomoxir-treated cells, reduced CSR and PCD may be the results of reduced cell proliferation (Supplementary Fig. 5e). These results collectively suggested that commitment of activated B cells to CSR and PCD is controlled by mitochondrial functions, as indicated by P1 and P2 populations, irrespective of specific metabolic pathways.

### Metabolic and gene expression profiles of P1 and P2 cells

**Figure 3:**
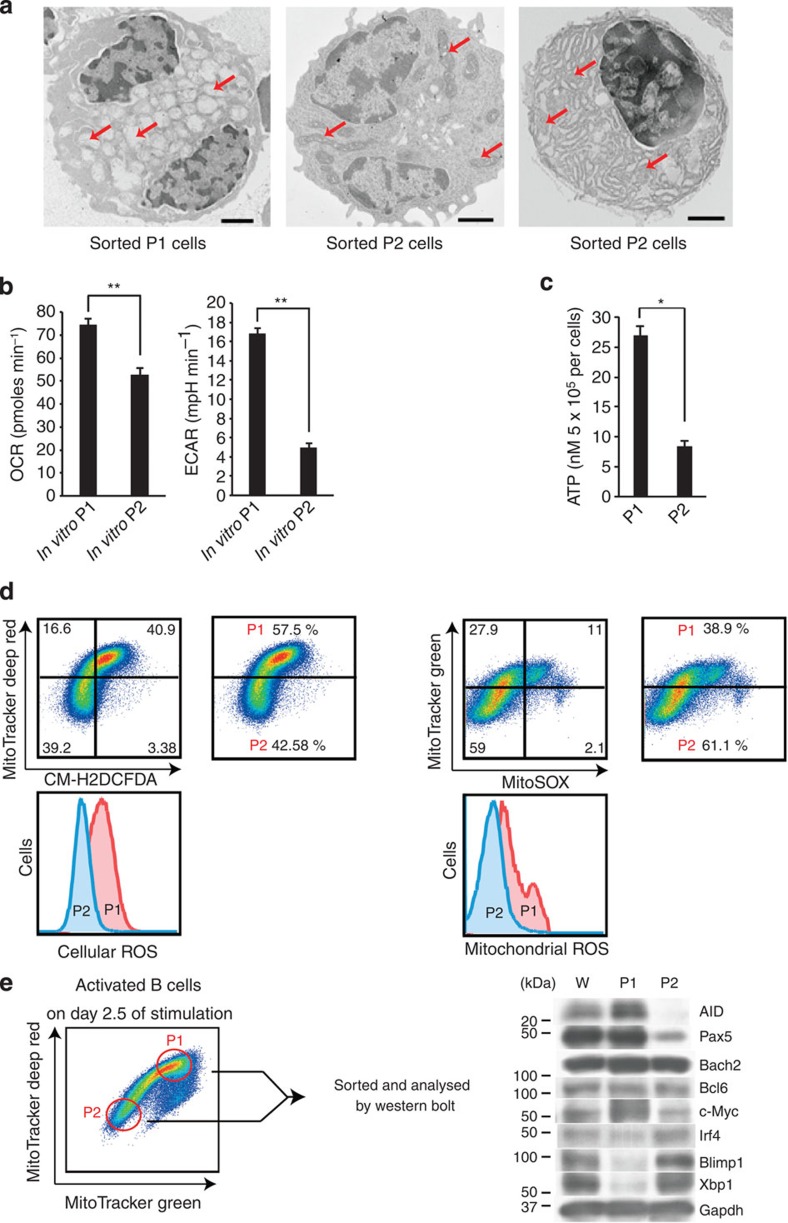
Characterization of P1 and P2 cell populations. (**a**) Transmission electron microscopy of FACS-sorted P1 and P2 cells. Red arrow indicates mitochondria. Mitochondria in P1 cells have been damaged during the sorting procedure. A larger endoplasmic reticulum in developing P2 cells is shown (right). Scale bar, 2 μm. (**b**) OCR and ECAR of sorted P1 and P2 cells. **P<0.005 (two-tailed Student's *t*-test). (**c**) ATP levels in sorted P1 and P2 cells. *P<0.05 (two-tailed Student's *t*-test). (**d**) Flow cytometric analysis of ROS and the mitochondrial status by MitoTracker, CM-H2DCFDA, and MitoSOX staining. (**e**) Western blotting analysis of unsorted (W) and sorted P1 and P2 cells. Data shown are representative of at least two independent experiments. Data are shown as mean±s.e.m.

To characterize cells in P1 and P2 cell populations, *in vitro*-activated P1 and P2 cells were investigated by transmission electron microscopy (Fig. 3a). Mitochondrial mass increased in P1 cells, consistent with the above results (Fig. 1). To further evaluate mitochondrial function, we determined the oxygen consumption rate (OCR) in P1 and P2 cells. Basal OCR, an indicator of oxidative phosphorylation, was 1.4 times higher in P1 than in P2 cells (Fig. 3b). The basal extracellular acidification rate (ECAR, a marker of glycolysis) was significantly higher in P1 than in P2 cells (Fig. 3b). ATP levels were consistently higher in P1 than in P2 cells (Fig. 3c). We also investigated the roles of OCR and ECAR during B-cell fate determination, using various reagents described in Fig. 2 (Supplementary Fig. 4b). However, there was no obvious involvement of OCR and ECAR in B-cell differentiation. Because mROS play key roles in various cell functions^35^,^39^,^40^, we evaluated their levels in P1 and P2 cell populations. We observed a ROS^high^ population in P1 cells (Fig. 3d and Supplementary Figs 1d and 2b,c). We also investigated the roles of ROS generation during B-cell fate determination using various reagents described in Fig. 2. ROS were induced by P1 cell-promoting reagents, whereas their levels were reduced by P2 cell-promoting reagents (Supplementary Figs 6–8). This finding suggested that ROS promoted CSR but suppressed PCD.

Protein levels in P1 cells were increased for Pax5, Bcl6, AID and c-Myc, which have been reported to promote CSR. Levels of Blimp1, Irf4 and Xbp1 proteins were higher in P2 than in P1 cells. The expression level of c-Myc increased in P1 cells, whereas that of Pax5 significantly decreased in P2 cells (Fig. 3e). These expression patterns were consistent with our hypothesis that P1 and P2 cells are cell populations precommitted to CSR and PCD.

### Differential haeme synthesis in P1 and P2 cell populations

**Figure 4:**
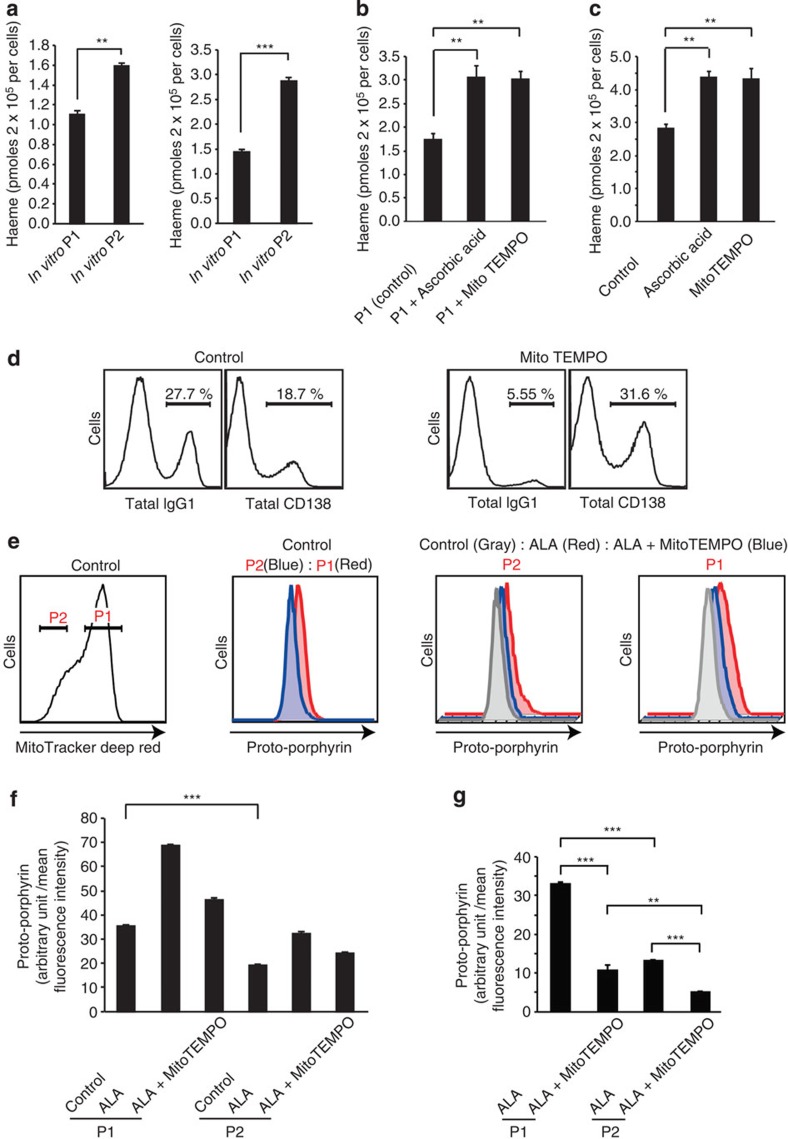
Differential haeme synthesis in P1 and P2 cell populations. (**a**) Haeme levels in sorted P1 and P2 cells from *in vitro*-activated B cells (left) or GC B cells in an immunized spleen (right). (**b**) Haeme levels in sorted P1 cells treated with indicated antioxidants. (**c**) Haeme levels in *in vitro*-activated B cells treated with indicated antioxidants. (**d**) Flow cytometric analysis of the differentiation status monitored by CD138 and IgG1 expression after 4 days of culture with LPS+IL-4 in the presence or absence of MitoTEMPO. (**e**) Flow cytometric analysis of PpIX fluorescence in P1 and P2 cells. *In vitro*-activated B cells treated with indicated reagents were assessed. (**f**) Mean fluorescence intensities (MFI) of PpIX as shown in (**e**) are plotted. (**g**) Increased MFI of PpIX after treatment with indicated reagents is plotted. Data shown are representative of three independent experiments. Data are shown as mean±s.e.m. **P<0.05, ***P<0.005. (two-tailed Student's *t*-test).

Because Bach2 promotes CSR and inhibits PCD via the repression of Blimp1 expression^21^,^41^, the reduced level of Bach2 correlates strongly with reciprocal Blimp1 expression in the normal course of B-cell activation. However, we found significant expression levels of both Blimp1 and Bach2 in P2 cells (Fig. 3e). Expression levels of Bach2 and Bcl6 were similar between P1 and P2 cells. We first determined the haeme levels that inhibit Bach2 function^36^. Haeme levels were higher in P2 cells than in P1 cells (Fig. 4a). *In vivo*-generated P2 cells also showed higher haeme levels than P1 cells (Fig. 4a). These results indicated a direct relationship between haeme levels and mitochondrial function. We then investigated the possible role of ROS in haeme synthesis. Haeme levels (Fig. 4b,c) were increased and PCD (Figs 2 and 4d and Supplementary Fig. 9) was promoted after treatment of P1 cells or activated B cells with ascorbic acid or MitoTEMPO, a mitochondria-targeted antioxidant. In MitoTEMPO treated cells, cellular ROS level was little reduced by day 4 (Supplementary Fig. 9c). We accordingly performed a time-course analysis of cellular ROS and found an antioxidant effect of MitoTEMPO on day 2 cells (24 h after MitoTEMPO treatment, Supplementary Fig. 9c). These data indicated that ROS at early time points was important in B-cell fate determination.

TPP cation is usually used as a carrier for targeting antioxidant to mitochondria. Because MitoTEMPO also uses TPP, TPP is a suitable control for assessing antioxidant function of MitoTEMPO. However, TPP was toxic to activated B cells, because cell numbers of TPP-treated cells decreased (Supplementary Fig. 10a). TPP, unlike MitoTEMPO, has no specific role in CSR and PCD, as shown in Supplementary Fig. 10b. TPP inhibited CSR and PCD in a dose-dependent manner. These results collectively suggested that mROS inhibited haeme synthesis and promoted CSR.

### mROS interfere with addition of ferrous ions to PpIX

The generation of 5-aminolevulinic acid (ALA) catalysed by ALA synthase is the initial step in haeme synthesis. As the final step, ferrous ion is incorporated into protoporphyrin IX (PpIX) by ferrochelatase (Fech). Haeme oxygenase-1 (HO-1) expression is induced by haeme to reduce the cellular haeme level. In contrast, CD71, the transferrin receptor, is required for iron uptake. The levels of haeme synthesis were not simply explained by expression levels of ALA synthase, CD71, or HO-1 (Supplementary Fig. 11). We accordingly investigated the processes of haeme synthesis affected by mROS by comparing the amounts of PpIX in the P1 and P2 cell populations. As shown in Fig. 4e, PpIX levels in P1 cells were higher than those in P2 cells, indicating that substrates and enzymes required to form the haeme precursor PpIX are not impaired but rather enhanced in the P1 cell population. We next assessed PpIX generation by the addition of ALA, whose synthesis is the rate-limiting step in haeme synthesis. PpIX levels were higher in P1 than in P2 cells, and this effect was counteracted by the treatment of P1 cells with MitoTEMPO (Fig. 4e–g). PpIX accumulation was simply explained by enhanced PpIX production or inhibition of the addition of ferrous ions to PpIX. The haeme level in P1 cells was low, and the antioxidant reversed the impairment of haeme synthesis (Fig. 4a–c). These data collectively suggested that mROS inhibited haeme synthesis by interfering with the addition of ferrous ions to PpIX. However, the precise mechanisms by which mROS inhibited haeme synthesis await further investigation.

### Haeme synthesis plays a key role in fate determination

**Figure 5:**
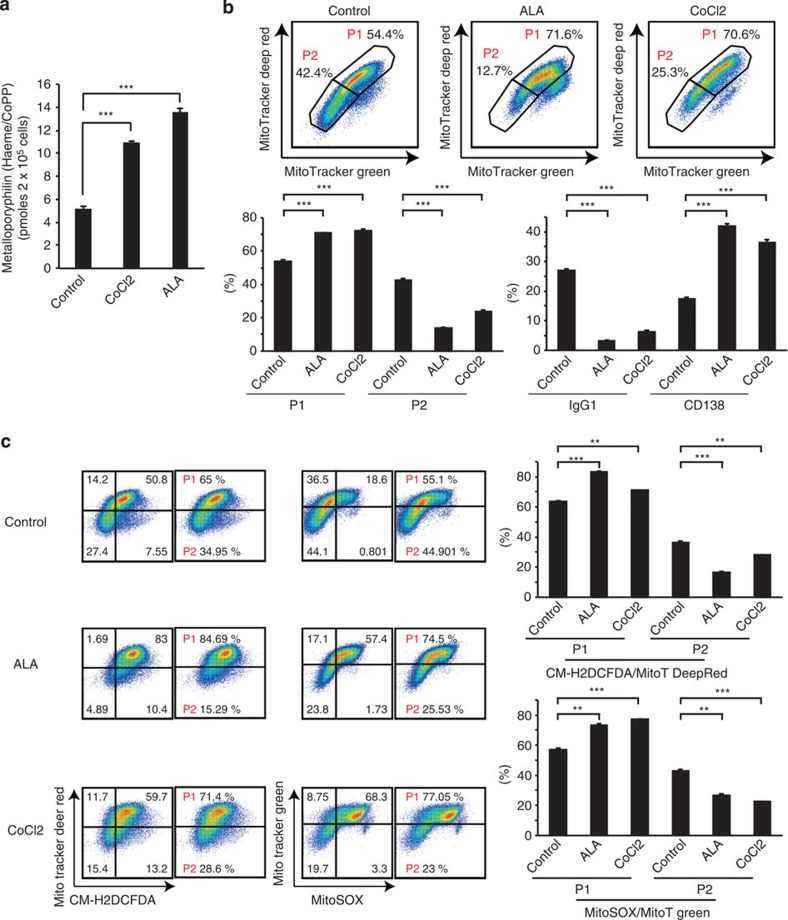
Differential production of ROS instructs cell fates in activated B cells. (**a**) Metalloporphyrin levels of *in vitro*-activated B cells treated with indicated reagents. Metalloporphyrin in control or ALA-treated cells consisted of haeme, whereas that in CoCl_2_-treated cells consisted of haeme and CoPP. ***P<0.005 (two-tailed Student's *t*-test). (**b**) Flow cytometric analysis of mitochondrial status monitored by MitoTracker staining and differentiation status monitored by CD138 and IgG1 expression on day 4. ***P<0.005 (two-tailed Student's *t*-test). (**c**) Flow cytometric analysis of mitochondrial status and cellular ROS monitored by MitoTracker DeepRed and CM-H2DCFDA (left) staining or mitochondrial status and mROS monitored by MitoTracker Green and MitoSOX (right) staining. **P<0.05, ***P<0.005. (two-tailed Student's *t*-test) FACS Data shown in **b**,**c** are representative of three independent experiments. Data are shown as mean±s.e.m.

To further confirm the role of *de novo* haeme synthesis in B-cell fate determination, we treated *in vitro*-activated B cells with ALA and CoCl_2_, a component of cobalt protoporphyrin (CoPP; not a substrate for HO-1 but an inhibitor of Bach function)^42^. Consistently with the increased haeme level after the addition of ALA (Fig. 5a), PCD was promoted despite the high ROS level (Fig. 5b,c). This response strongly indicated that haeme synthesis itself, rather than ROS, plays a key role in PCD. This interpretation was further supported by the finding that PCD was enhanced after the treatment of activated B cells with CoCl_2_, which increases the amounts of metalloporphyrin, a molecule consisting of haeme and CoPP, and ROS level (Fig. 5a–c).

### Bach2 inhibits P2 cell generation

**Figure 6:**
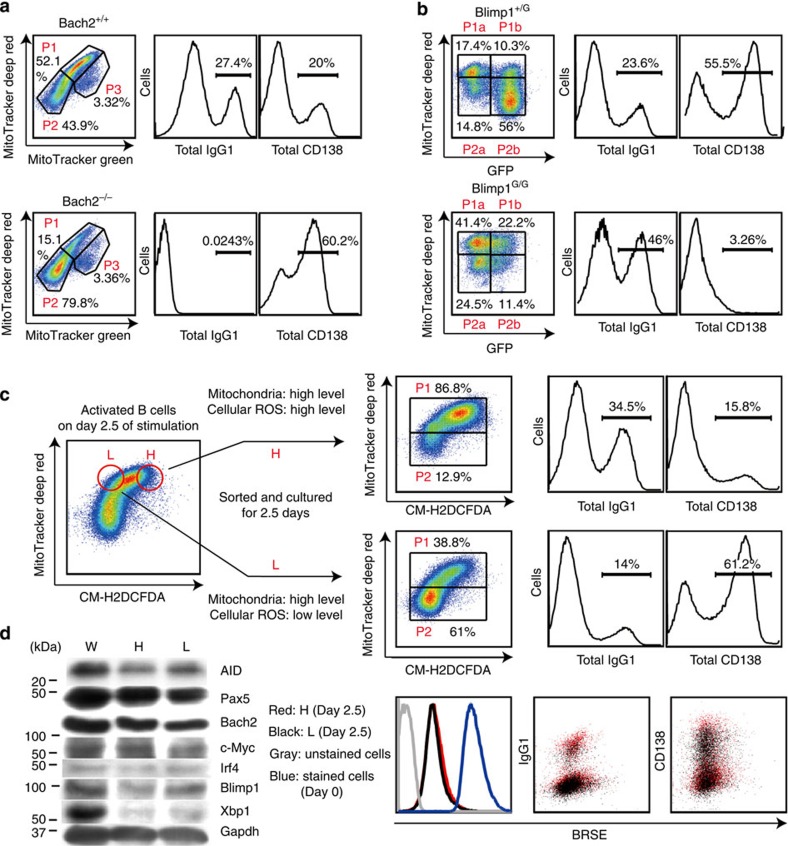
Differential ROS production provides instructive signals for fates of activated B cells. (**a**) Flow cytometric analysis of mitochondrial membrane potential and size monitored by MitoTracker staining (left) or differentiation of B cells monitored by CD138 and IgG1 expression (middle and right) in LPS+IL-4-stimulated Bach2^−/−^ or WT B cells. (**b**) Flow cytometric analysis of mitochondrial membrane potential monitored by MitoTracker staining and GFP expression (left) or differentiation of B cells monitored by CD138 and IgG1 expression (middle and right) in LPS+IL-4-stimulated Blimp1^G/G^ or Blimp1^G/+^ B cells. (**c**) Diagrammatic representation of experimental protocol. Flow cytometric analysis of differentiation of sorted ROS^low^(L) and ROS^high^(H) cells (top and middle) and proliferation (bottom). (**d**) Western blotting analysis of unsorted (W) and sorted ROS^l^°^w^(L) and ROS^high^(H) cells. Data shown are representative of at least two independent experiments.

We next examined the effects of Bach2 on mitochondrial status, because the mROS–haeme relationship was based on modulating Bach2 function in P1 and P2 cells. As shown in Fig. 6a, more P2 cells were generated from Bach2^−/−^ B cells than from wild-type cells, consistent with the observation that P2 cells contain more haeme than do P1 cells (Fig. 4a). These findings supported the notion that Bach2 function was suppressed in P2 cells and indicated that Bach2 was required for both P1 cells and CSR. Because Blimp1 expression is increased in Bach2^−/−^ B cells, we next investigated whether Blimp1 is required for P2 cell generation, using Blimp1-green fluorescent protein (GFP) knock-in mice. In these mice, *Blimp1* was replaced with GFP, so that GFP expression was under the control of endogenous *Blimp1* regulatory elements, indicating that Blimp1-expressing cells was easily identified by GFP expression. Because committed cells to PCD, but not to CSR, express Blimp1, GFP^+^ cells was PC-committed populations. P2 cells were generated from Blimp1-expressing cells (GFP^+^ cells in Blimp1^+/GFP^ B cells, Fig. 6b upper column) and could not be observed in Blimp1^GFP/GFP^ B cells (Fig. 6b lower column). These data indicated that P2 cells were generated in a manner depending on Blimp1 function and suggested that the mitochondrial status of P2 cells were a physiological property of differentiated plasma cells.

### Differential ROS production provides instructive signals

**Figure 7:**
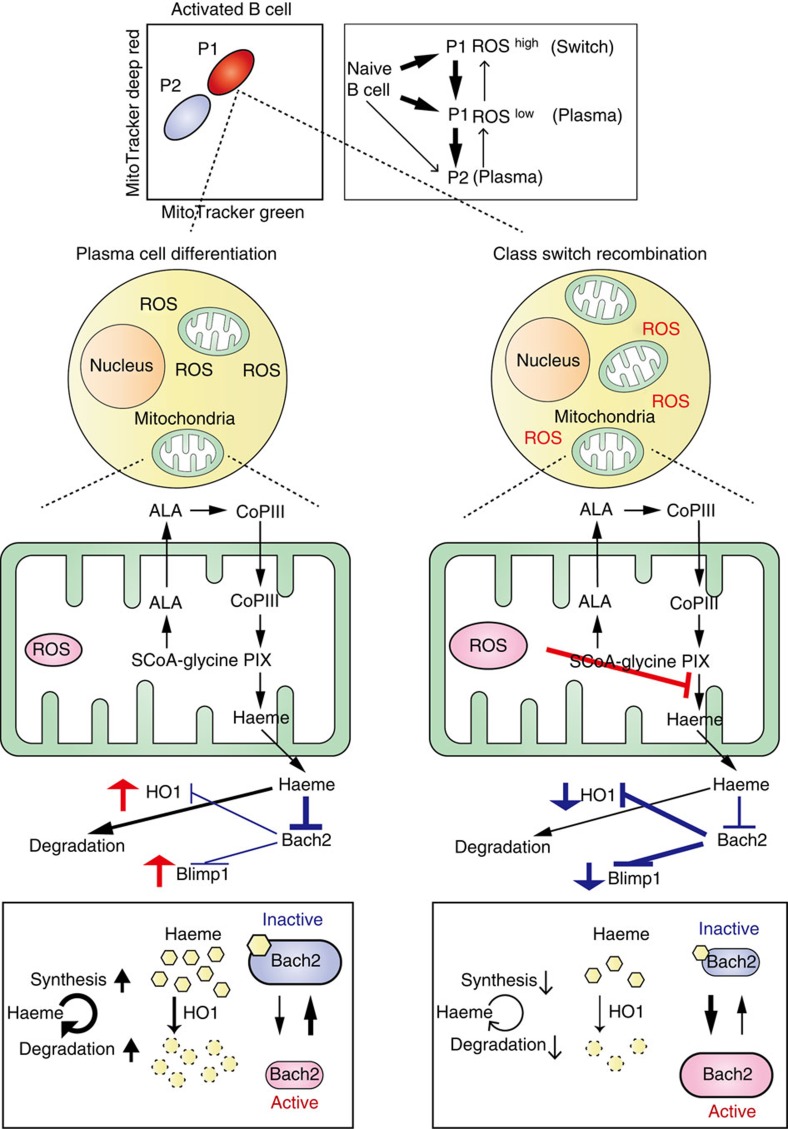
Schematic representation of models of B-cell differentiation during normal course of immune reaction. On activation, B cells acquire mitochondrial mass and membrane potential (and are called P1 cells) and proliferate. Stochastic differences in mitochondrial activity within P1 cells generate diverse P1 cells having various ROS amounts (in this scheme, they are simply classified into two groups, P1 ROS^high^ and P1 ROS^low^ cells). In addition to the signalling function, excess amounts of ROS have another function of attenuating haeme synthesis. In general, newly generated haeme that cannot bind to haeme proteins is degraded immediately by HO-1. Haeme that binds to high- but not to low-affinity haeme-binding proteins (such as Bach2), are retained for a longer period. Thus, the effects of haeme on the regulation of low-affinity haeme-binding proteins are limited. Moreover, increasing amounts of free haeme induce HO-1 expression, which enhances the activity of free haeme clearance. Accordingly, the level of effective haeme concentration that regulates low-affinity haeme-binding proteins cannot be achieved by transient elevation of haeme contents. In P1 ROS^low^ cells, the upregulation of haeme turnover, which supports a continuous haeme supply, inhibits Bach2 function and promotes the PCD program (from P1 ROS^low^ to P2 cells). Theoretically, initial changes might occur at any steps described in this scheme. For example, HO-1-dependent degradation of haeme provides an antioxidant, which can cause reduction of ROS level. The important steps in the respective immune reactions await identification. The majority of naïve B cells are also P2 cells, but almost all the cells show increased mitochondrial mass, membrane potential (Fig. 1a) and Bach2 protein expression after activation (not shown). Accordingly, P1 and P2 populations observed in naïve B cells may be different from P1 and P2 populations of activated B cells.

As shown in Fig. 1c, commitment of activated B cells to CSR or PCD was predicted by their mitochondrial status. P1 cells, which contained higher mitochondrial mass and membrane potential, preferentially underwent CSR, whereas P2 cells, which contained lower mitochondrial mass and membrane potential, differentiated into plasma cells. Bach2 function is required for CSR, and reduced activity of Bach2 promotes PCD. However, the expression level of Bach2 differed little between P1 and P2 cells (Fig. 3e). Thus, the differential regulation of Bach2 activity is key in these cell fate-determination processes. Bach2 function was maintained by inhibition of haeme synthesis by mROS produced in P1 cells (Fig. 4a–g). In contrast, Bach2 function was inhibited in P2 cells, allowing the expression of Bach2 target genes such as Blimp1 (Fig. 3e). P2 cells appear to arise from plasma cell-committed populations (Figs 3e and 6b). To determine whether the same scenario is applicable in the initial step of cell fate determination, we evaluated the differentiation capacity of ROS^high^ and ROS^low^ cells within P1 cells. Between ROS^high^ and ROS^low^ cells derived from the P1 cell population, we found no differences in protein levels of transcription factors essential to CSR and PCD (Fig. 6d). These results suggested that cells at this stage are not committed to either differentiation pathway. When undifferentiated ROS^high^ and ROS^low^ cells were collected and cultured for an additional 2.5 days, ROS^high^ cells underwent predominantly CSR, whereas ROS^low^ cells differentiated into plasma cells (Fig. 6c). In addition, the preferential appearance of P1 cells was observed in cultured ROS^high^ cells, but appearance of P2 cells was observed in cultured in ROS^low^ cells (Fig. 6c). Because CSR and PCD are influenced by cell proliferation, numbers of cell divisions were assessed by BRSE. As shown in Fig. 6c, there was little difference in the cell division numbers between these populations. These results indicated that change in mROS generation was the cause of the cell fate decision after B-cell activation. These findings together indicate that fate determination of activated B cells is dependent on ROS level, which negatively regulates haeme synthesis. Halflife of Bach2 protein was longer than that of Pax5 protein, because significant amount of Bach2 protein was observed in 5 days-cultured B cells (Supplementary Fig. 12). These data indicated that quantitative regulation of haeme plays an important role in promoting PCD. In other words, PCD is a reversible state before loss of Bach2 protein. Our results show the mechanism of induction of differentiation in the lineage commitment process of B cells (Fig. 7). In general, commitment to specific lineages of haematopoietic cells is an integrated interpretation of instructive and stochastic signals^14^,^15^,^16^,^17^,^43^,^44^. Precise understanding of the mechanism of B-cell fate determination requires further identification of the mechanisms by which levels of ROS and haeme synthesis are regulated by instructive signals and by which haeme functions as a signalling molecule.

## Discussion

Fate determination of activated B cells toward CSR or PCD appears to be an intracellular stochastic process^14^,^15^. However, the underlying stochastic changes resulting in the initiation of either pathway are incompletely understood.

In this study, we identified mROS, which reflect individual mitochondrial status, as a signal for the initiation of fate determination of activated B cells. mROS promoted CSR and suppressed PCD by attenuating haeme synthesis because haeme inhibits Bach2 function^36^. The underlying mechanism of the inhibition of haeme synthesis is unknown. According to our combined experimental results, mROS inhibited the addition of ferrous ions to PpIX in mitochondria. There are at least three ways by which this inhibition may occur—mROS may inhibit haeme synthesis by inhibiting Fech activity; mROS may inhibit the reduction of ferric ion to ferrous ion, a substrate of Fech, in mitochondria; or there may be a defect in iron availability in mitochondria. In this regard, involvement of Fech in CSR and PCD was examined *in vivo*. As shown in Supplementary Fig. 13a, CSR was enhanced in mice treated with Fech inhibitor, but its *P* value did not meet the criteria of statistical significance. Additionally, mitochondrial function in activated B-cell fate determination was investigated *in vivo*. Enhanced CSR and reduced PCD were observed in complexI/III-inhibitor-treated mice (Supplementary Fig. 13b).

B cells, having high affinity to B-cell receptors (BCR) and antigens, differentiate preferentially into plasma cells, indicating that antigen recognition strength is an instructive signal for PCD. PI3K and Akt activities affect activated B-cell fates^38^. The PI3K–Akt pathway, downstream of BCR signalling, inhibits CSR by repressing AID gene expression via Foxo1 and Foxo3a phosphorylation, and promotes PCD by inducing Blimp1 expression. However, the mechanism by which PI3K–Akt induces Blimp1 is unknown. Another downstream signal of BCR, Erk, is also required for PCD. In this case, Blimp1 expression is induced by Erk-dependent Elk1 phosphorylation^45^. Irf4 is required for both CSR and PCD^12^,^13^. The antigen-binding affinity of BCR regulates the magnitude of Irf4 expression and acts as a dose-dependent regulator of activated B-cell fates^12^,^13^. A weaker BCR signal induces a lower Irf4 level, resulting in CSR. In contrast, increased antigen affinity augments BCR signalling-mediated Irf4 expression and promotes PCD. Dose-dependent cell fate determination by Irf4 is achieved via discrimination between the affinities of various target sequences, as suggested previously^7^,^46^. Lower-affinity recognition sequences of Irf4 are occupied by Irf4 in differentiating plasma cells, which promote PCD by inducing the gene sets essential for PCD^12^. In T cells, Irf4 expression is also controlled by T-cell receptor affinity^47^, indicating the presence of a similar regulatory network in T cells. Thus, a relationship between the amount of Irf4 and cell fate determination has been clearly demonstrated. However, the mechanisms by which the strength of BCR signalling is sensed and Irf4 expression is regulated are unknown.

Inhibition of the PI3K–Akt signal strongly promotes P1 cell generation, accompanied by reduced PCD and enhanced CSR. In other words, the PI3K–Akt signal, a downstream signal of BCR, promotes P2 cell generation, which in turn promotes PCD. An increased amount of mROS is produced in the P1 cell population, attenuating haeme synthesis. Bach2 function is maintained in the P1 cell population and suppressed in the P2 cell population because Bach2 is negatively regulated by haeme. In differentiating plasma cells, increasing amounts of haeme inhibit Bach2 function and in turn promote Blimp1 expression because Blimp1 is a direct target of Bach2 in B cells. Recent reports have shown that Irf4 is a direct target of Bach2 in macrophages^48^ or regulatory T cells^49^, indicating that reduced Bach2 function in the P2 cell population is responsible for the induction of Irf4 gene expression. Alternatively, the involvement of another transcriptional repressor also regulated by haeme is an attractive mechanism by which Irf4 expression may be induced in haeme-rich P2 cells. Mitf is a strong candidate as a transcriptional repressor of Irf4 in B cells^50^, and contains three Cys-Pro (CP) motifs involved in haeme binding. The involvement of Mitf in this haeme-decoding system awaits further elucidation.

In summary, we provide a model to explain how instructive and stochastic signals of activated B cells are integrated via changes in mitochondrial function that modulate mROS levels. Besides the function of mROS as signals for various cell fate determinations, mROS attenuate haeme synthesis, and the amount of haeme also plays a key role in cell fate determination of activated B cells by regulating the activities of haeme-binding transcription factors including Bach2. Accumulating evidence indicates that self-renewal and differentiation of HSCs are dependent on mROS, although the precise role of mROS in these processes is unclear. Thus, our findings provide insight into the fundamental roles of haeme synthesis, which is regulated by mitochondrial status, in cell fate determination processes. Identification of mROS as inhibitors of haeme synthesis has important implications for understanding not only B cells but also HSC biology, and may be applicable to the treatment of hematopoietic disorders.

## Methods

### Mice

This study received ethics approval from the Institutional Review Board of Kyoto University (reference number, Med Kyo13086). Bach2^−/−^ (provided by Dr Igarashi, Tohoku University), Blimp1-GFP (provided by Dr Nutt, The Walter and Eliza Hall Institute of Medical Research) and wild-type mice on C57BL/6 genetic background were maintained in a specific pathogen-free mouse facility, and male mice were used at 8–12 weeks of age. Procedures involving animals and their care followed the guidelines for animal treatment of the Institute of Laboratory Animals, Kyoto University.

### Cell preparation and *in vitro* culture

Naïve B cells were prepared by magnetic cell sorting using anti-CD43 microbeads (130-49-801; Miltenyi Biotec). Purified splenic B cells (5 × 10^5^ cells ml^−1^) were cultured in RPMI 1640 medium (11875-093; Gibco) supplemented with 10% fetal bovine serum (FBS) and 2-mercaptoethanol (50 μM; Nacalai Tesque) in the presence of LPS (40 μg ml^−1^; L7136/015K4074/014K4112/086K4056 and L3755/102M4018V; Sigma) and IL-4 (20 ng ml^−1^; R&D systems) or anti-mouse CD40 (HM40-3) antibody (1 μg ml^−1^; 553721; BD PharMingen) and IL-4 for 4 days. In some experiments, cells cultured for 2.5 days were sorted by flow cytometry using FACSAria (Becton Dickinson) and restimulated with LPS+IL-4 in the presence of the indicated reagents (Figs 1c and 6d).

### CFSE or BRSE labelling

Purified splenic naïve B cells (5 × 10^6^ cells) were stained with 500 μl of 5-μM CFSE solution (345-06441; DOJINDO) in a CO_2_ incubator at 37 °C for 10 min. Sorted cells were stained with 500 μl of 5-μM BRSE solution (D-2219; Molecular Probes) in a CO_2_ incubator at 37 °C for 10 min.

### Flow cytometric analysis and cell sorting

After cultured cells were washed with prewarmed no-glucose RPMI 1640 medium (11879020; Gibco) supplemented with 10% FBS (staining buffer), 1.5 × 10^6^ cells were resuspended in 1 ml of staining buffer containing MitoTracker Green (20 nM; M7514; Invitrogen), MitoTracker DeepRed (20 nM; M22426; Invitrogen), MitoSOX (5 μM; M36008; Invitrogen), CM-H2DCFDA (5 μM; C6827; Invitrogen), TMRM (40 nM; 9105; Immunochemistry Technologies) or CellROX DeepRed (5 μM; C10422; Life Technologies) and incubated in a CO_2_ incubator at 37 °C for 30–40 min. Stained cells were washed with 1 ml of prewarmed staining buffer and used for FACS (fluorescence-activated cell sorting) analysis, cell sorting or further staining with antibodies. Normal rabbit serum (10%) containing Fc Block (2.4G2; BD PharMingen) was used as blocking reagent. For immunized spleen cells, 5 × 10^6^ cells were incubated with 1 ml of staining buffer containing MitoTracker Green (40 nM) and DeepRed (40 nM), and acid wash treatment was applied to remove surface-bound immunoglobulin before antibody staining. MitoTracker-stained 5 × 10^6^ spleen cells were suspended in 10 μl of acid solution (equal volumes of cold 0.1 M acetic acid, pH 4 and solution containing 0.17 M NaCl and 0.01 M KCl mixed before use), incubated on ice for 1 min and neutralized by the addition of 1 ml of cold Tris buffer (0.03 M Tris, pH 7.4, 0.8 × PBS, 2% FCS). The following antibodies (1/300 diluted) were used for staining: biotin-labelled anti-mouse IgG1 (553441; BD PharMingen), phycoerythrin (PE)-conjugated anti-mouse CD138 (553714; BD PharMingen), Brilliant Violet 421-conjugated anti-mouse CD138 (562610; BD PharMingen), PE/Cy7-conjugated anti-mouse B220 (552772; BD PharMingen), PE-conjugated anti-mouse FAS (554258; BD PharMingen), streptavidin–Brilliant Violet 421-conjugated (405226; Biolegend) and streptavidin–Brilliant Violet 570-conjugated (405227; Biolegend). For sorting immunized spleen cells, T-cell depletion was performed using IMAG and anti-Thy1.2 beads (551518; BD Biosciences) before staining with MitoTracker. Cytometric analysis and sorting of *in vitro* cultured and immunized cells was performed using FACSAria.

### Immunization

For T-cell-dependent immune response, NP-chicken gamma globulin (100 μg; NP-CGG; Biosearch Technologies) in complete Freund's adjuvant (Difco) was intraperitoneally injected. For T-cell-independent immune response, NP-Ficoll (50 μg) in PBS was intraperitoneally injected.

### Reagents used *in vivo*

During T-cell-independent immune reaction, *N*-methyl protoporphyrin (16 g kg^−1^ per day; MPP; Fech inhibitor; NMP576; Frontier Scientific) or *N*-benzylguanidine acetate (10 g kg^−1^ per day; NBA; complexes I, III inhibitor; SC-279892; SantaCruz) were intraperitoneally injected.

### Reagents used in *in vitro* culture

The following reagents were added to *in vitro* culture from days 2.5 to 4: methyl pyruvate (10 mM; 371173; Sigma), methyl malate (5 mM; 355-17971; Wako), etomoxir (90 μM; E1905; Sigma), rotenone (120 nM; R8875; Sigma), metformin (1 mM; 150959; SIGMA), oligomycin (1 nM; O4876; Sigma), 2-deoxy-D-glucose (200 μM; 154-17-6; SIGMA), haemin (30 μM; H9039; Sigma), ascorbic acid (200 μM; A7506; Sigma), MitoTEMPO (64 μM; ALX-430-150; Enzo Life Science) and TPP (64 μM; 309567; SIGMA); from days 1 to 4: LY294002 (3 μM; 1667; Biovision), AZD5363 (5 μM; S8019; Selleckchem) and 5-ALA hydrochloride (450 μM; 3785; Sigma); and from days 3 to 4: cobalt (II) chloride (150 μM; 232696; Sigma).

For OCR and ECAR assay, all the reagents were added at 36 h of culturing and incubated for 12 h. Exposing duration of cells to reagents was therefore from 36 to 48 h of culturing.

### Extracellular flux analysis

OCR and ECAR were measured in XF media (unbuffered RPMI 1640 containing 1% FBS, 11 mM glucose, 2 mM glutamine and 1 mM pyruvate) under basal conditions and in response to 1 μM oligomycin (O4876; Sigma), 1 μM carbonyl cyanide 4-(trifluoromethoxy)phenylhydrazone and 1 μM antimycin A (A0149; Sigma) on the XF96 extracellular flux analyzer (Seahorse Bioscience, Billerica, MA, USA). Basal OCR was calculated by subtraction of the residual rate after antimycin A treatment. After culture with LPS and IL-4 for 2.5 days, B cells were stained with MitoTracker Green and DeepRed and sorted using FACSAria. Sorted cells were cultured for 24 h under the same conditions to allow recovery from the stress of sorting. These cells were resuspended in XF media and were plated onto Seahorse cell plates (1 × 10^5^ cells per well) coated with poly-L-lysine hydrobromide (P2636; Sigma).

To assess the effects of reagents on ECAR and OCR, we prepared cells treated with various reagents under the conditions described in ‘Reagents used in *in vitro* culture.' After culture with LPS and IL-4 for 48 h, cells were resuspended in XF media and were plated onto Seahorse cell plates (1 × 10^5^ cells per well) coated with poly-L-lysine hydrobromide (P2636; Sigma). OCR and ECAR were measured as described above.

### Protein extraction and western blot analysis

Proteins were extracted with lysis buffer (Tri-HCl (pH 7.4), 150 mM NaCl, 1 mM EDTA, 1% NP-40, 0.5% NaDOC, 0.1% SDS, 1 mM Na_3_VO_4_, 1 mM phenylmethylsulphonyl fluoride, 1 μg ml^−1^ pepstatin, 1 μg ml^−1^ aprotinine and 10 μg ml^−1^ leupeptine) for 20 min on ice, and protein amounts were quantified using Micro BCA Protein Assay Reagent (23235; Pierce Chemical Co.). Whole-cell lysates (40 μg per lane) were resolved on SDS–polyacrylamide gels, electrotransferred onto polyvinylidene difluoride membranes and examined by immunoblot analysis. The primary antibodies used were anti-PAX5 (610862; BD PharMingen), anti-BCL6 (#4242; Cell Signaling), anti-c-MYC (NB600-302C; Novus Biologicals), anti-IRF4 (sc-6059; Santa Cruz), anti-BLIMP1 (NB600-235; Novus Biologicals), anti-XBP1 (sc-7160; Santa Cruz), anti-HO-1 (NBP1-97507; Novus Biologicals), anti-AID (provided by Dr Alt) and anti-GAPDH (#2118; Cell Signaling). Full-sized scans of all western blots shown in Figs 3 and 6 and Supplementary Figs 11 and 12 are provided in Supplementary Fig. 14. Quantification of intensity was performed using an Image J (http://imagej.nih.gov/ij/).

### Transmission electron microscopy

Sorted P1 and P2 cells (from B cells cultured for 3 days) were plated onto a Lab-Tek Chamber Slide (177445; Nunc), coated with poly-L-lysine hydrobromide and fixed with 4% paraformaldehyde/2% glutaraldehyde. Sections were viewed with a Hitachi H-7650 transmission electron microscope.

### Reverse transcription–PCR

Total RNAs were extracted from cultured B cells with TRIzol reagent (Gibco). Oligo (dT)-primed complementary DNAs were prepared with reverse transcriptase. Complementary DNAs were subjected to PCR reactions with the following primer pairs: Bach2 forward, 5′- CGCTGTCGAAAGAGGAAGCTGGAC -3′; Bach2 reverse, 5′- CCTGGATCTGCTCTGGACTCTGGA -3′; Pax5 forward, 5′- ATTGTCACAGGCCGAGACT -3′; Pax5 reverse, 5′- GCTGCAGGGCTGTAATAGT -3′; Gapdh forward, 5′- CCATCACCATCTTCCAGGA -3′; Gapdh reverse, 5′- CCTGCTTCACCACCTTCTT -3′.

### Haeme quantification

Sorted or cultured B cells (2 × 10^5^) were suspended in 500 μl of saturated oxalic acid in water and heated at 121 °C for 20 min. Emission peaks at 608 and 662 nm using 400 nm excitation were measured (Hitachi MPF-4).

### Intracellular measurement of PpIX fluorescence

Intracellular PpIX fluorescence was evaluated by FACSAria (excitation 405 nm; emission, 625/20 nm filter).

### Intracellular ATP quantification

ATP was measured using an ATP determination kit (A22066; Invitrogen) according to the manufacturer's instructions.

### Statistical analysis

OCR, ECAR, ATP, protoporphyrin, haeme and metalloporphyrin amounts were compared by Student's *t*-test. All reported *P* values were two-tailed and *P* values <0.05 were considered to indicate significance.

## Author contributions

K.-J.J., H.M., A.M. and M.S. performed the experiments; A.M., K.A., T.H., Y.N., K.T., K.I. and S.T. assisted with the experiments; K.I. and S.L.N. provided the mice; M.S. designed and conceptualized the study; Y.N. performed the statistical analysis; Y.N., K.A., S.T., S.L.N. and K.I. made comments; A.S., K.-J.J., S.T., K.I. and M.S. interpreted the data; and M.S. wrote the manuscript.

## Additional information

**How to cite this article:** Jang, K.-J. *et al*. Mitochondrial function provides instructive signals for activation-induced B-cell fates. *Nat. Commun.* 6:6750 doi: 10.1038/ncomms7750 (2015).

## Supplementary Material

Supplementary Information Supplementary Figures 1-14
