# Supplementary material for: Mitochondrial function provides instructive signals for activation-induced B-cell fates
Source: Nat Commun. 2015 Apr 10;6:6750. doi: 10.1038/ncomms7750 (PMC4403446; doi:10.1038/ncomms7750)
Supplement: Supplementary Information — Supplementary Figures 1-14 [file ncomms7750-s1.pdf]

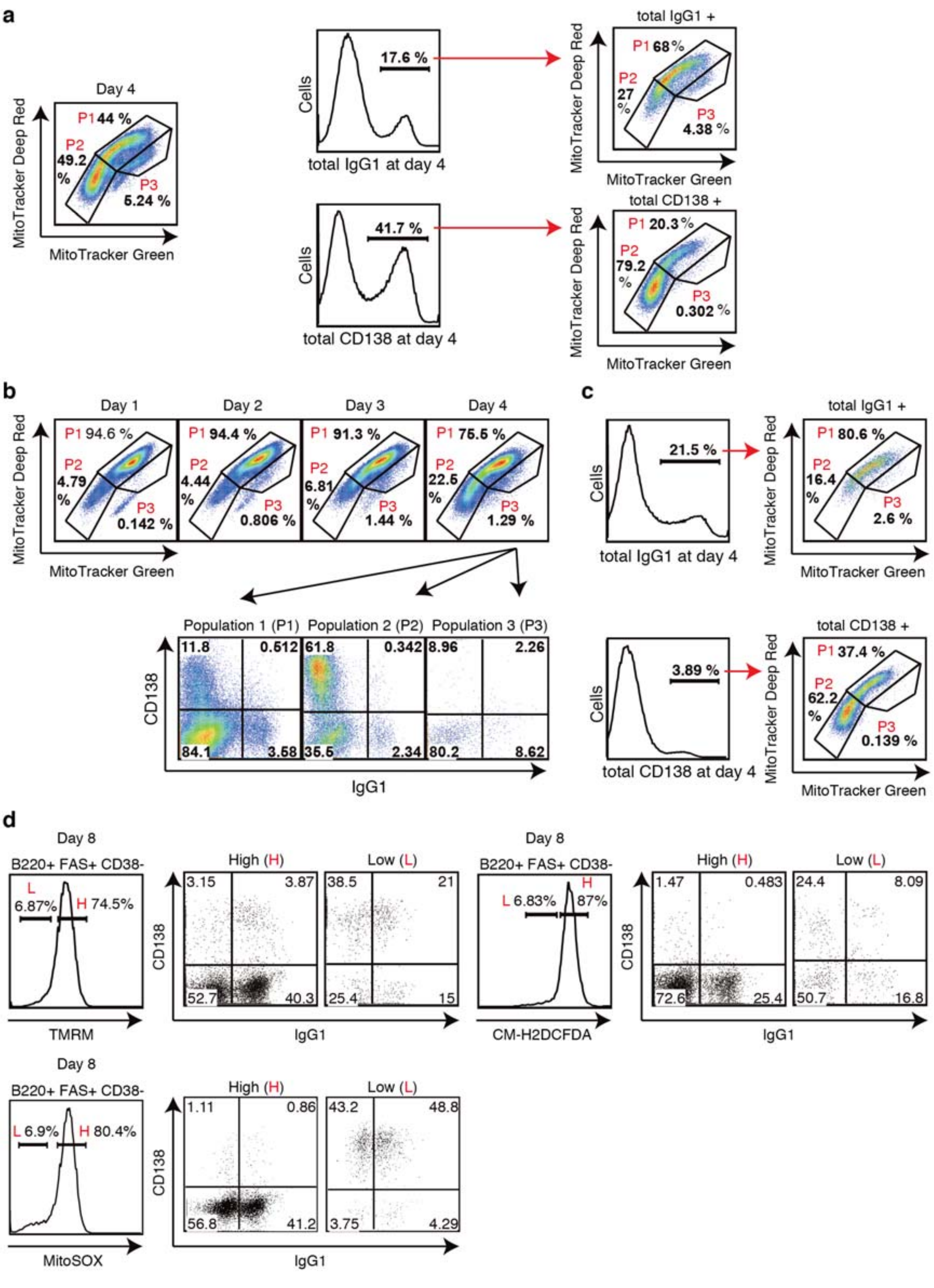
a
Day 4
P1 44 %
P2
49.2
%
P3
5.24 %
MitoTracker Deep Red
MitoTracker Green
17.6 %
Cells
total IgG1 at day 4
41.7 %
Cells
total CD138 at day 4
total IgG1 +
P1 68 %
P2
27
%
P3
4.38 %
total CD138 +
P1 20.3 %
P2
79.2
%
P3
0.302 %
b
Day 1
Day 2
Day 3
Day 4
P1 94.6 %
P2
4.79
%
P3
0.142 %
P1 94.4 %
P2
4.44
%
P3
0.806 %
P1 91.3 %
P2
6.81
%
P3
1.44 %
P1 75.5 %
P2
22.5
%
P3
1.29 %
Population 1 (P1)
Population 2 (P2)
Population 3 (P3)
11.8
0.512
84.1
3.58
61.8
0.342
35.5
2.34
8.96
2.26
80.2
8.62
CD138
IgG1
c
21.5 %
total IgG1 at day 4
total IgG1 +
P1 80.6 %
P2
16.4
%
P3
2.6 %
3.89 %
total CD138 at day 4
total CD138 +
P1 37.4 %
P2
62.2
%
P3
0.139 %
d
Day 8
B220+ FAS+ CD38-
L
6.87%
H 74.5%
TMRM
High (H)
Low (L)
3.15
3.87
52.7
40.3
38.5
21
25.4
15
IgG1
Day 8
B220+ FAS+ CD38-
H
L 6.83%
87%
CM-H2DCFDA
High (H)
Low (L)
1.47
0.483
72.6
25.4
24.4
8.09
50.7
16.8
IgG1
Day 8
B220+ FAS+ CD38-
L 6.9%
H 80.4%
MitoSOX
High (H)
Low (L)
1.11
0.86
56.8
41.2
43.2
48.8
3.75
4.29
IgG1

Supplementary Figure 1 Activated B cells are subdivided into three groups according to mitochondrial status

(a) Flow cytometric analysis of mitochondrial status monitored by MitoTracker staining or differentiation monitored by CD138 and IgG1 expression on day 4 in LPS + IL-4-stimulated B cells. **(b, c)** Flow cytometric analysis of mitochondrial status monitored by MitoTracker staining (**b** top, **c**) or differentiation monitored by CD138 and IgG1 expression on day 4 (**b** bottom, **c**) in α-CD40 + IL-4-stimulated B cells. **(d)** Flow cytometric analysis of cellular ROS (CM-H2DCFDA), mitochondrial ROS (MitoSOX), and mitochondrial membrane potential (TMRM) on day 8 after immunization with NP-CGG and the differentiation status of indicated populations in germinal-center B cells ($B220^{+}CD38^{-}FAS^{+}$). Data shown are representative of three independent experiments.

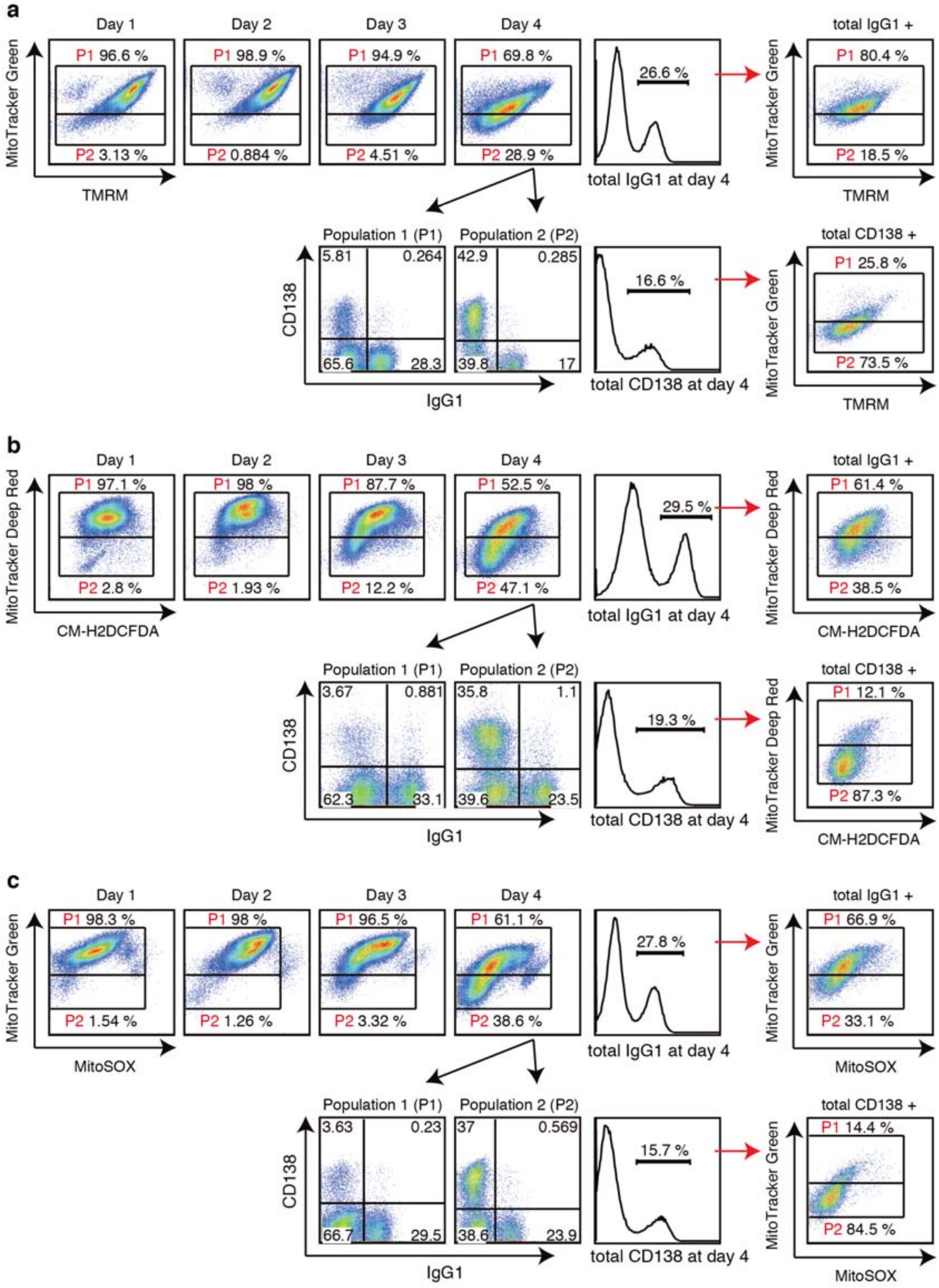
a
Day 1
Day 2
Day 3
Day 4
total IgG1 +
MitoTracker Green
TMRM
P1 96.6 %
P2 3.13 %
P1 98.9 %
P2 0.884 %
P1 94.9 %
P2 4.51 %
P1 69.8 %
P2 28.9 %
26.6 %
total IgG1 at day 4
P1 80.4 %
P2 18.5 %
Population 1 (P1)
Population 2 (P2)
CD138
IgG1
5.81
0.264
65.6
28.3
42.9
0.285
39.8
17
16.6 %
total CD138 at day 4
total CD138 +
P1 25.8 %
P2 73.5 %
b
MitoTracker Deep Red
CM-H2DCFDA
P1 97.1 %
P2 2.8 %
P1 98 %
P2 1.93 %
P1 87.7 %
P2 12.2 %
P1 52.5 %
P2 47.1 %
29.5 %
P1 61.4 %
P2 38.5 %
3.67
0.881
62.3
33.1
35.8
1.1
39.6
23.5
19.3 %
P1 12.1 %
P2 87.3 %
c
MitoSOX
P1 98.3 %
P2 1.54 %
P1 98 %
P2 1.26 %
P1 96.5 %
P2 3.32 %
P1 61.1 %
P2 38.6 %
27.8 %
P1 66.9 %
P2 33.1 %
3.63
0.23
66.7
29.5
37
0.569
38.6
23.9
15.7 %
P1 14.4 %
P2 84.5 %

Supplementary Figure 2 Mitochondrial mass, membrane potential, ROS and cellular ROS in Activated B cells

(a) Flow cytometric analysis of mitochondrial status monitored by MitoTracker Green and TMRM staining on the indicated day or differentiation monitored by CD138 and IgG1 expression on day 4 in LPS + IL-4-stimulated B cells. **(b)** Flow cytometric analysis of mitochondrial mass and mitochondrial ROS monitored by MitoTracker Green and MitoSOX staining on the indicated day or differentiation monitored by CD138 and IgG1 expression on day 4 in LPS + IL-4-stimulated B cells. **(c)** Flow cytometric analysis of mitochondrial membrane potential and cellular ROS monitored by MitoTracker Green and CM-H2DCFDA staining on the indicated day or differentiation monitored by CD138 and IgG1 expression on day 4 in LPS + IL-4-stimulated B cells. Data shown are representative of three independent experiments.

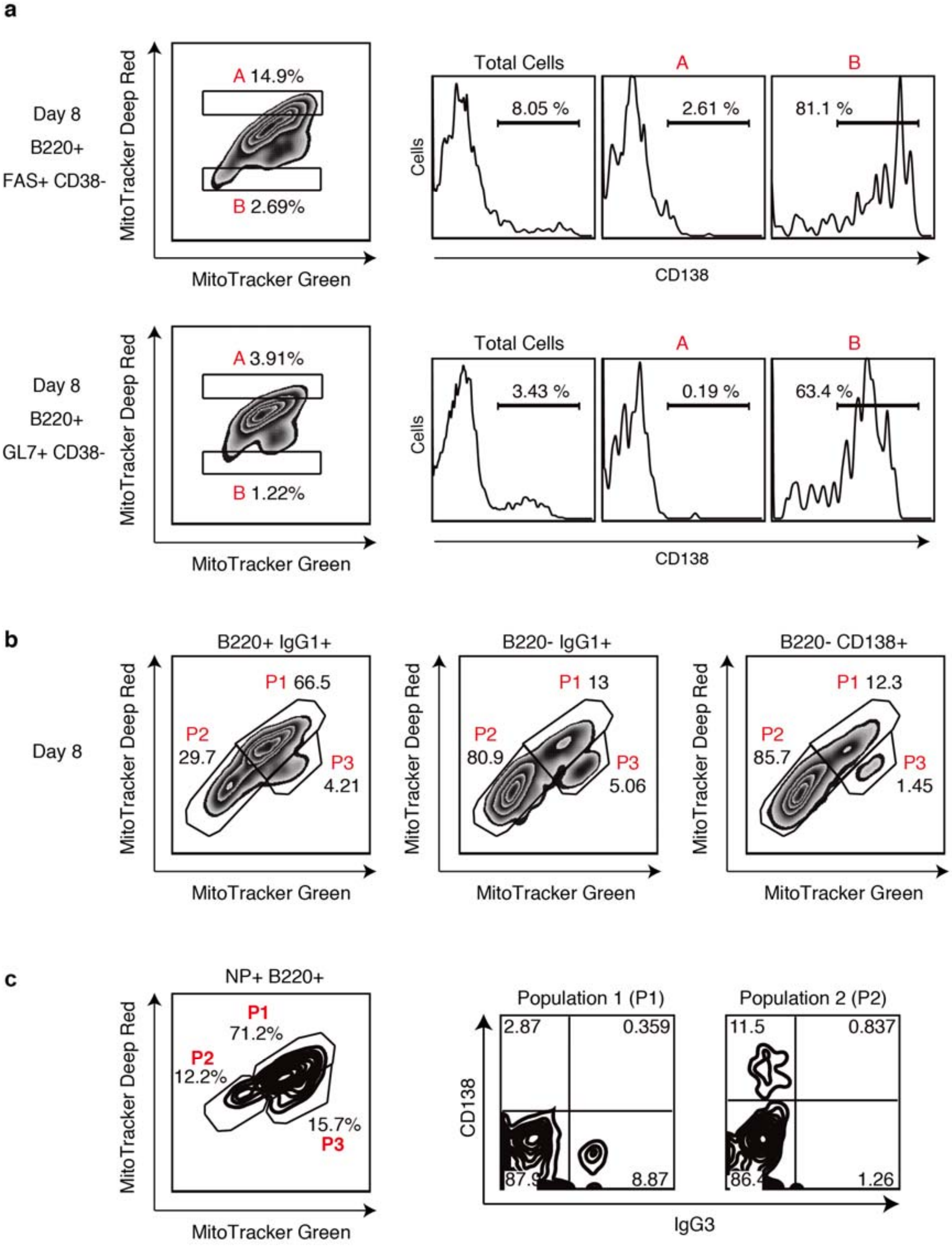
a
Day 8
B220+
FAS+ CD38-
MitoTracker Deep Red
MitoTracker Green
A 14.9%
B 2.69%
Total Cells
A
B
Cells
8.05 %
2.61 %
81.1 %
CD138
Day 8
B220+
GL7+ CD38-
A 3.91%
B 1.22%
3.43 %
0.19 %
63.4 %
b
Day 8
B220+ IgG1+
P1 66.5
P2 29.7
P3 4.21
B220- IgG1+
P1 13
P2 80.9
P3 5.06
B220- CD138+
P1 12.3
P2 85.7
P3 1.45
c
NP+ B220+
P1 71.2%
P2 12.2%
15.7% P3
Population 1 (P1)
2.87
0.359
8.87
Population 2 (P2)
11.5
0.837
1.26
CD138
IgG3

Supplementary Figure 3 Association of mitochondrial status and activated B cell fates *in vivo*

(a) Flow cytometric analysis of mitochondrial status monitored by MitoTracker staining (left) and PCD monitored by CD138 expression (right) in indicated populations. Spleen cells from the same mouse were stained with indicated antibodies. **(b)** Flow cytometric analysis of mitochondrial status monitored by MitoTracker staining in indicated B cell subsets. **(c)** Flow cytometric analysis of mitochondrial status (left) of $B220^{+}NP^{+}$ cells from the spleen 3 days after immunization with NP-Ficoll and the differentiation status of population 1 (middle) and population 2 (right). Data shown are representative of three independent experiments.

a

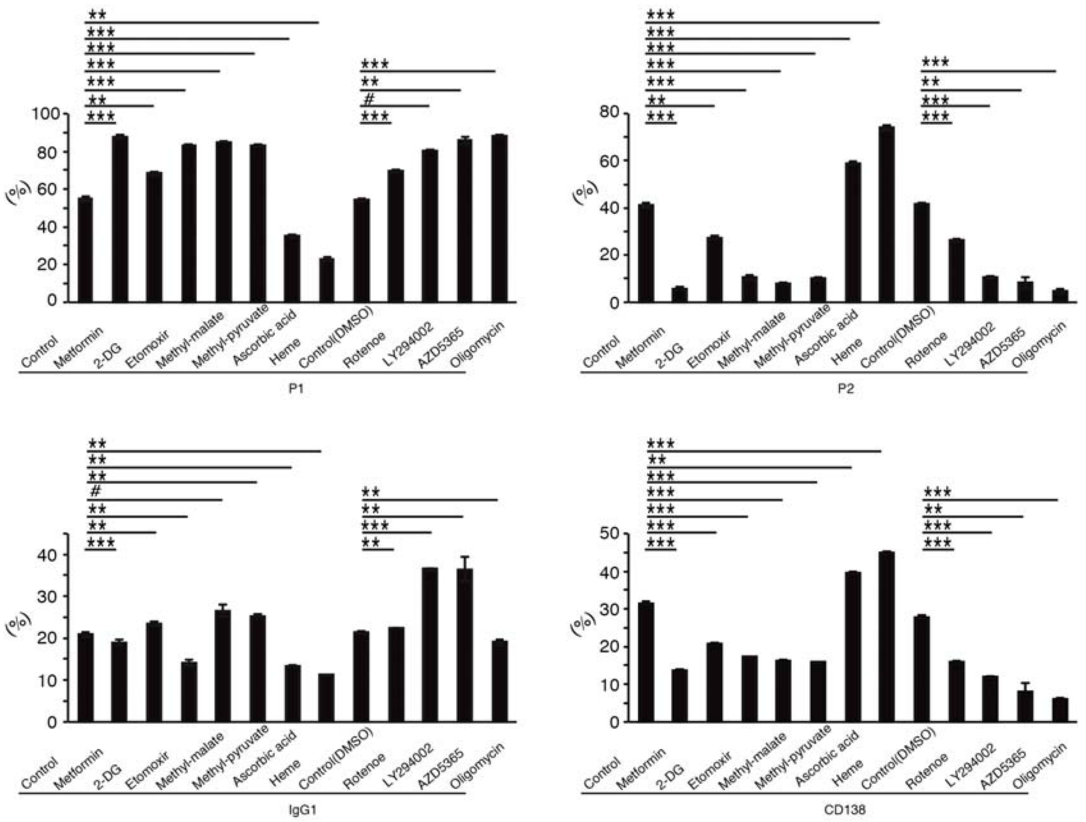
(%)
Control
Metformin
2-DG
Etomoxir
Methyl-malate
Methyl-pyruvate
Ascorbic acid
Heme
Control(DMSO)
Rotenoe
LY294002
AZD5365
Oligomycin
P1
P2
IgG1
CD138

b

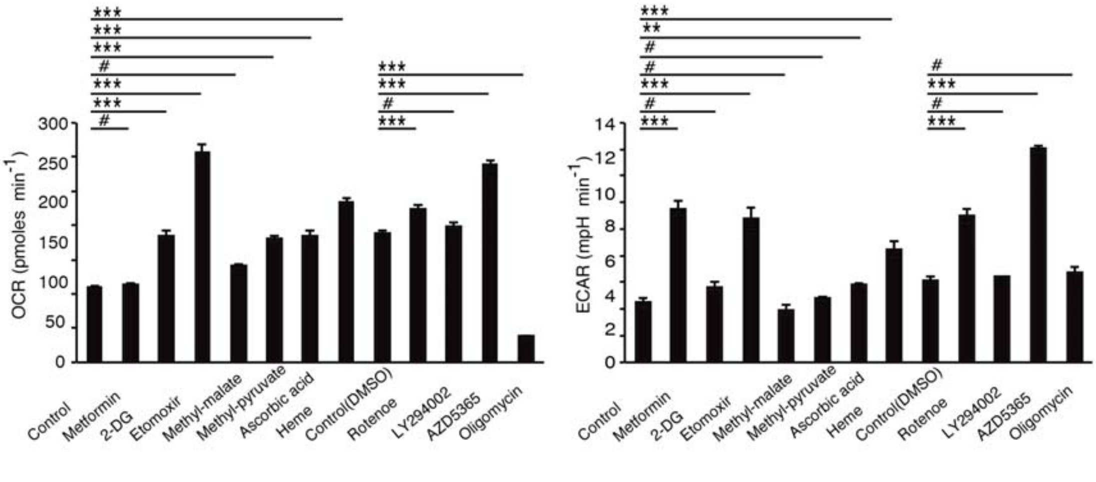
OCR (pmoles min^{-1})
ECAR (mpH min^{-1})
Control
Metformin
2-DG
Etomoxir
Methyl-malate
Methyl-pyruvate
Ascorbic acid
Heme
Control(DMSO)
Rotenoe
LY294002
AZD5365
Oligomycin

Supplementary Figure 4 Association of mitochondrial status with activated B cell fate *in vitro*

(a) Flow cytometric analysis of mitochondrial status monitored by MitoTracker staining or differentiation monitored by CD138 and IgG1 expression after 4 days of culture with LPS + IL-4 in the presence or absence of the indicated reagents are summarized. **(b)** ECAR and OCR of activated B cells treated with indicated reagents are summarized. Data are shown as mean ± s.e.m. #$p > 0.05$ (not significant), **$p < 0.05$, ***$p < 0.005$. (two-tailed Student's *t*-test)

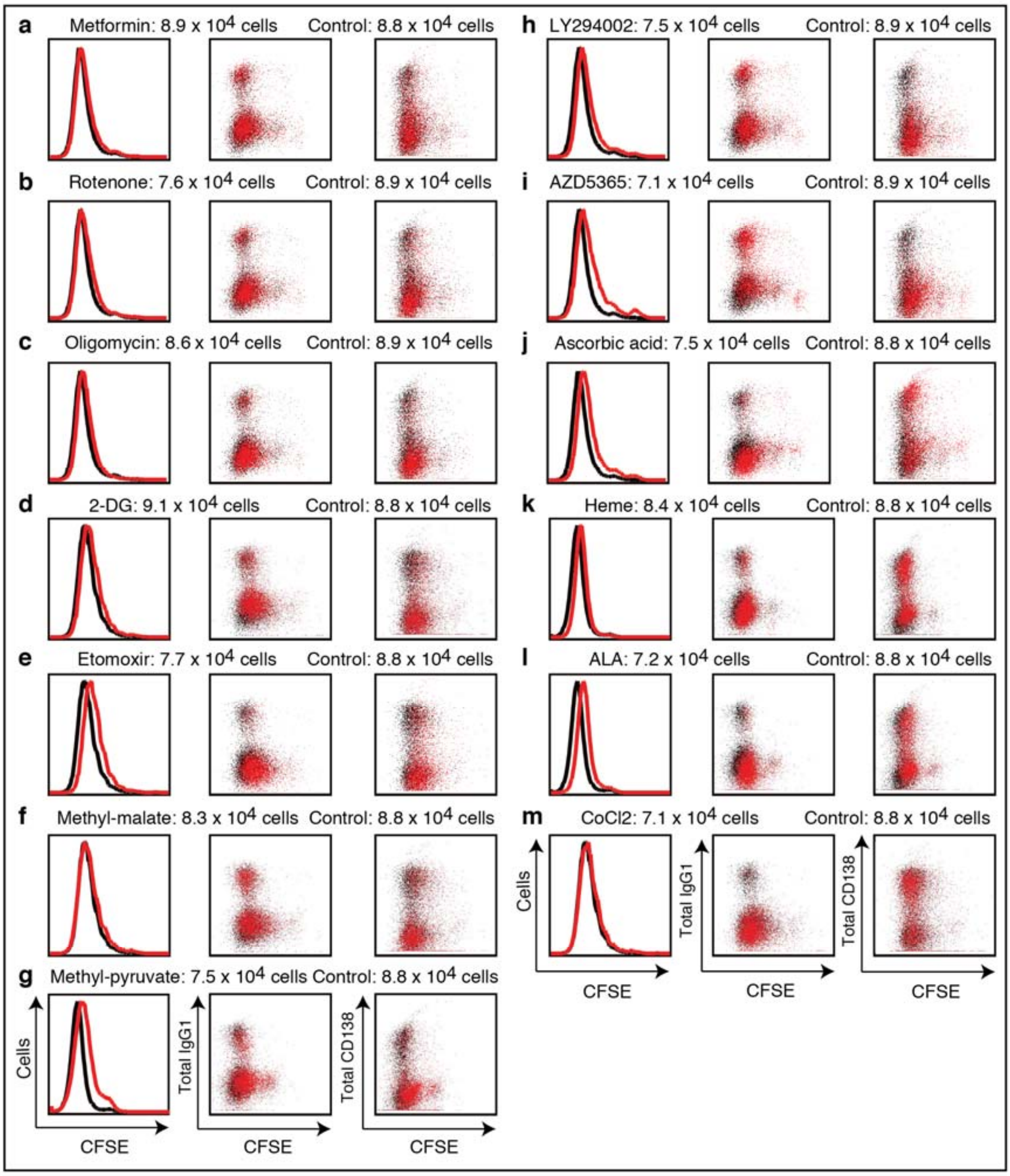

a Metformin: 8.9 x 10^4 cells Control: 8.8 x 10^4 cells
b Rotenone: 7.6 x 10^4 cells Control: 8.9 x 10^4 cells
c Oligomycin: 8.6 x 10^4 cells Control: 8.9 x 10^4 cells
d 2-DG: 9.1 x 10^4 cells Control: 8.8 x 10^4 cells
e Etomoxir: 7.7 x 10^4 cells Control: 8.8 x 10^4 cells
f Methyl-malate: 8.3 x 10^4 cells Control: 8.8 x 10^4 cells
g Methyl-pyruvate: 7.5 x 10^4 cells Control: 8.8 x 10^4 cells
h LY294002: 7.5 x 10^4 cells Control: 8.9 x 10^4 cells
i AZD5365: 7.1 x 10^4 cells Control: 8.9 x 10^4 cells
j Ascorbic acid: 7.5 x 10^4 cells Control: 8.8 x 10^4 cells
k Heme: 8.4 x 10^4 cells Control: 8.8 x 10^4 cells
l ALA: 7.2 x 10^4 cells Control: 8.8 x 10^4 cells
m CoCl2: 7.1 x 10^4 cells Control: 8.8 x 10^4 cells
Cells
Total IgG1
Total CD138
CFSE

Supplementary Figure 5 Relationship between cell proliferation and various reagents in differentiating B cells

Flow cytometric analysis of cell proliferation monitored by CFSE staining or differentiation monitored by CD138 and IgG1 expression after 4 days of culture with LPS + IL-4 in the presence (red) or absence (black) of the indicated reagents. Data shown are representative of three independent experiments.

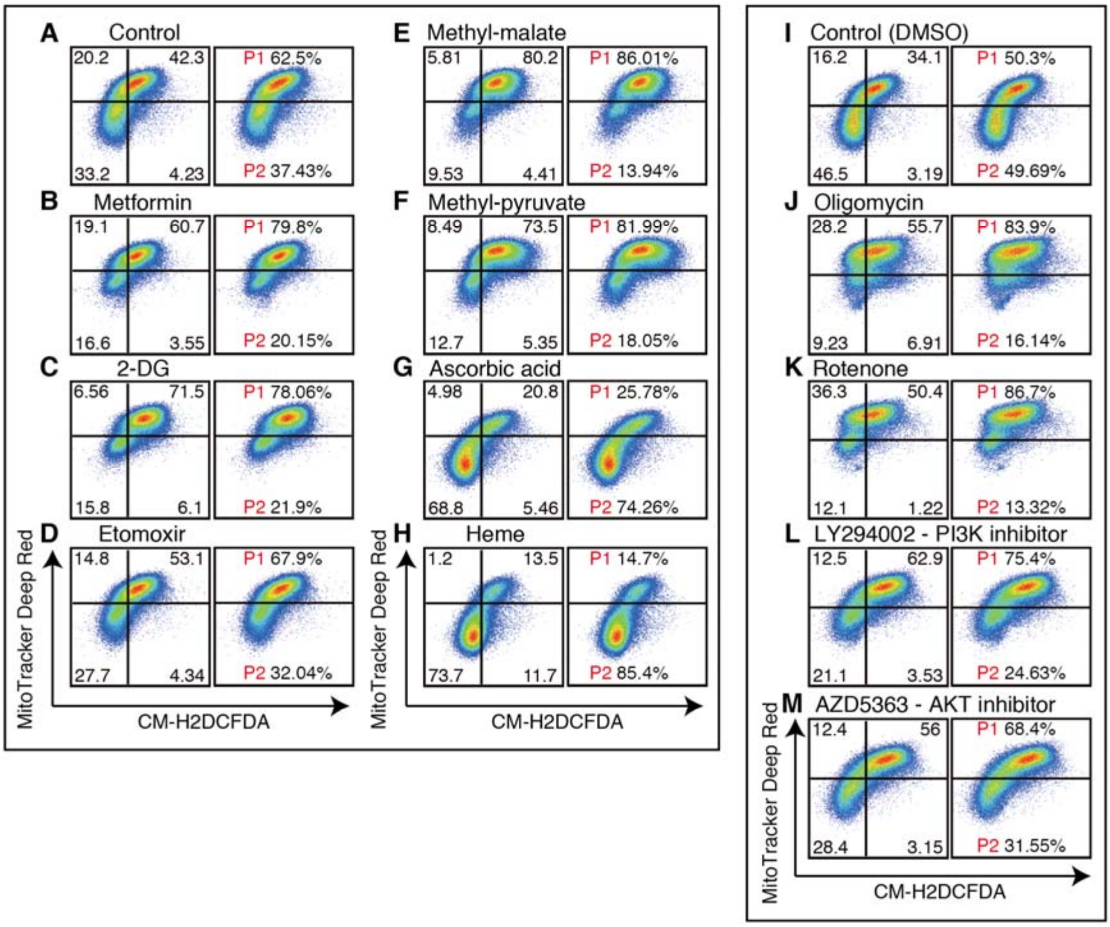

Supplementary Figure 6 Relationship between cellular ROS and various reagents in differentiating B cells

Flow cytometric analysis of mitochondrial status and cellular ROS monitored by staining with MitoTracker DeepRed and CM-H2DCFDA, a cellular ROS indicator, after 4 days of culture with LPS + IL-4 in the presence or absence of the indicated reagents. Data shown are representative of three independent experiments.

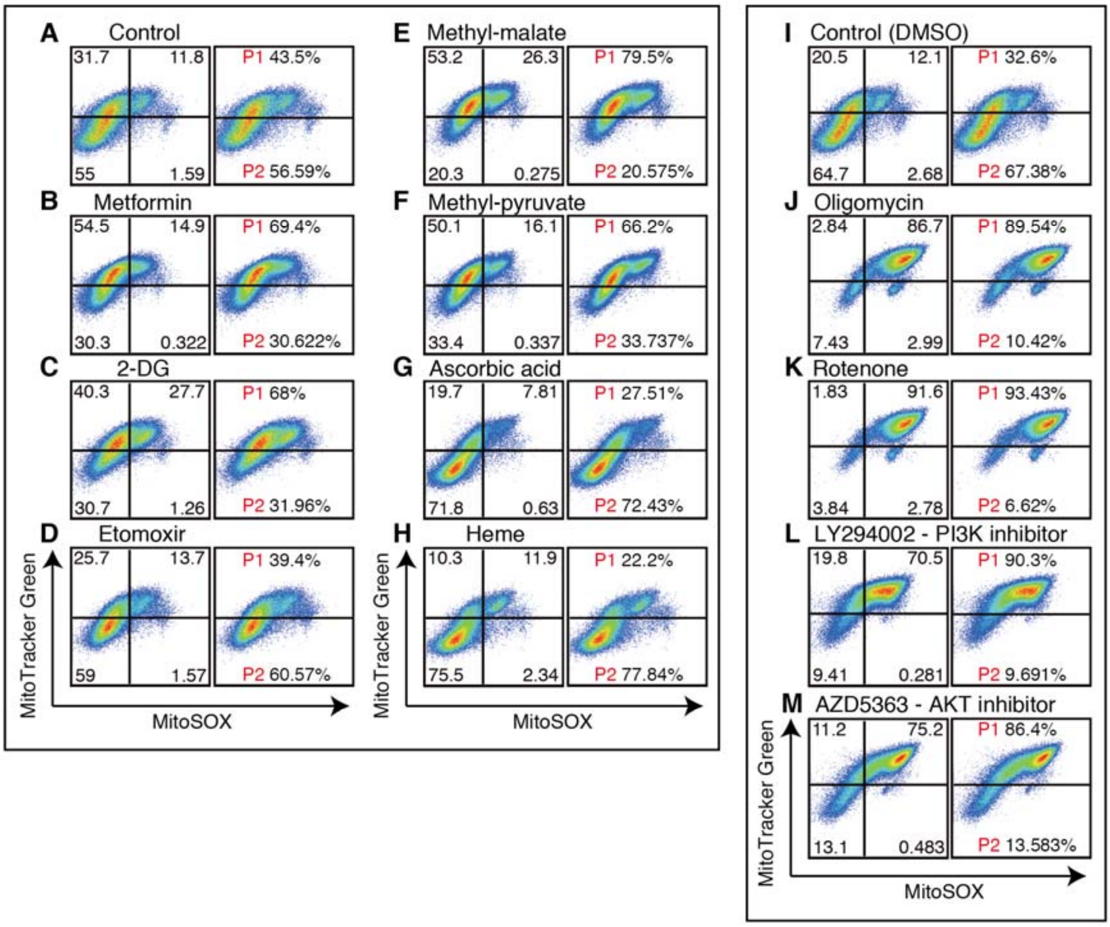

Supplementary Figure 7 Relationship between mitochondrial ROS and various reagents in differentiating B cells

Flow cytometric analysis of mitochondrial status and mROS monitored by MitoTracker Green and MitoSOX staining after 4 days of culture with LPS + IL-4 in the presence or absence of the indicated reagents. Data shown are representative of three independent experiments.

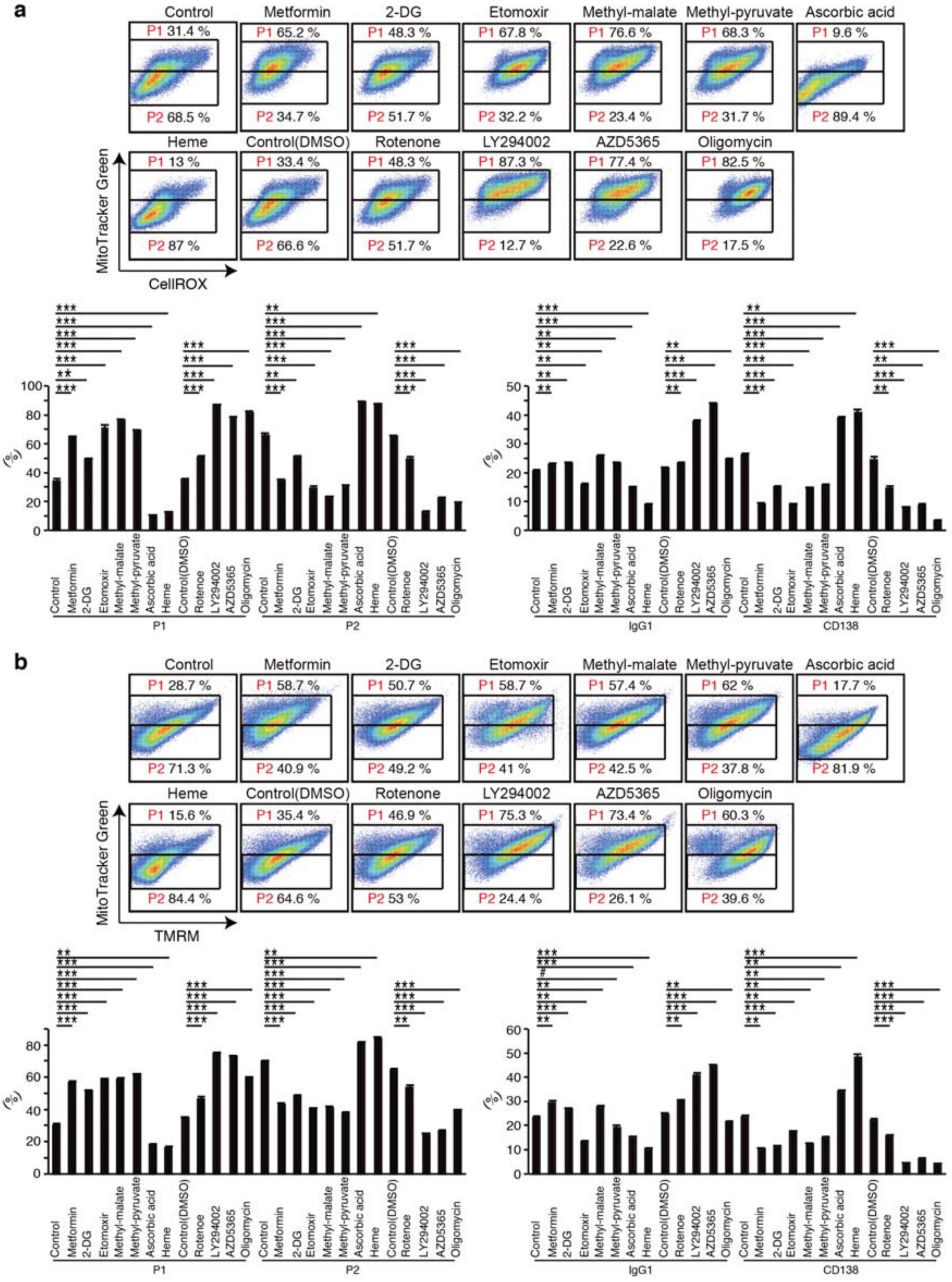
a
Control
Metformin
2-DG
Etomoxir
Methyl-malate
Methyl-pyruvate
Ascorbic acid
P1 31.4 %
P2 68.5 %
P1 65.2 %
P2 34.7 %
P1 48.3 %
P2 51.7 %
P1 67.8 %
P2 32.2 %
P1 76.6 %
P2 23.4 %
P1 68.3 %
P2 31.7 %
P1 9.6 %
P2 89.4 %
Heme
Control(DMSO)
Rotenone
LY294002
AZD5365
Oligomycin
P1 13 %
P2 87 %
P1 33.4 %
P2 66.6 %
P1 48.3 %
P2 51.7 %
P1 87.3 %
P2 12.7 %
P1 77.4 %
P2 22.6 %
P1 82.5 %
P2 17.5 %
MitoTracker Green
CellROX
(%)
P1
P2
IgG1
CD138
b
Control
Metformin
2-DG
Etomoxir
Methyl-malate
Methyl-pyruvate
Ascorbic acid
P1 28.7 %
P2 71.3 %
P1 58.7 %
P2 40.9 %
P1 50.7 %
P2 49.2 %
P1 58.7 %
P2 41 %
P1 57.4 %
P2 42.5 %
P1 62 %
P2 37.8 %
P1 17.7 %
P2 81.9 %
Heme
Control(DMSO)
Rotenone
LY294002
AZD5365
Oligomycin
P1 15.6 %
P2 84.4 %
P1 35.4 %
P2 64.6 %
P1 46.9 %
P2 53 %
P1 75.3 %
P2 24.4 %
P1 73.4 %
P2 26.1 %
P1 60.3 %
P2 39.6 %
MitoTracker Green
TMRM
(%)
P1
P2
IgG1
CD138

Supplementary Figure 8 Association of mitochondrial status with activated B cell fate *in vitro*

(a) Flow cytometric analysis of mitochondrial mass and cellular ROS monitored by MitoTracker Green and CellROX staining or differentiation monitored by CD138 and IgG1 expression after 4 days of culture with LPS + IL-4 in the presence or absence of the indicated reagents are summarized. **(b)** Flow cytometric analysis of mitochondrial mass and mitochondrial membrane potential monitored by MitoTracker Green and TMRM staining or differentiation monitored by CD138 and IgG1 expression after 4 days of culture with LPS + IL-4 in the presence or absence of the indicated reagents are summarized. Data are shown as mean ± s.e.m. $^{**}p < 0.05$, $^{***}p < 0.005$. (two-tailed Student's *t*-test)

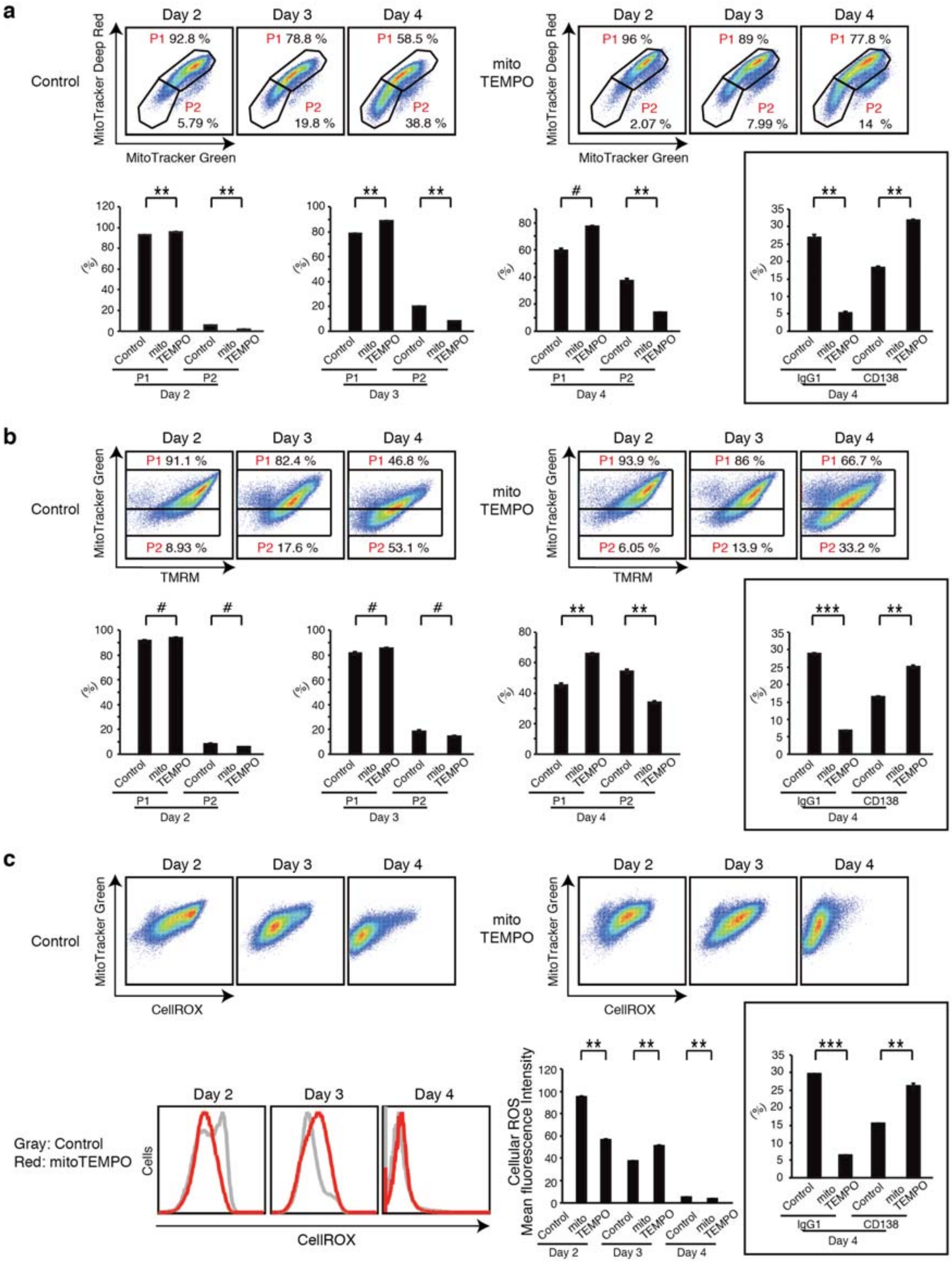
a
Control
mito TEMPO
Day 2
Day 3
Day 4
MitoTracker Deep Red
MitoTracker Green
P1 92.8 %
P2 5.79 %
P1 78.8 %
P2 19.8 %
P1 58.5 %
P2 38.8 %
P1 96 %
P2 2.07 %
P1 89 %
P2 7.99 %
P1 77.8 %
P2 14 %
(%)
Control
mito TEMPO
P1
P2
IgG1
CD138
**
#
b
MitoTracker Green
TMRM
P1 91.1 %
P2 8.93 %
P1 82.4 %
P2 17.6 %
P1 46.8 %
P2 53.1 %
P1 93.9 %
P2 6.05 %
P1 86 %
P2 13.9 %
P1 66.7 %
P2 33.2 %
***
c
CellROX
Gray: Control
Red: mitoTEMPO
Cells
Cellular ROS
Mean fluorescence Intensity

Supplementary Figure 9 Effect of MitoTEMPO on activated B cell fate determination

(a) Flow cytometric analysis of mitochondrial mass and mitochondrial membrane potential monitored by MitoTracker Green and DeepRed staining on the indicated day or differentiation monitored by CD138 and IgG1 expression after 4 days of culture with LPS + IL-4 in the presence or absence of the indicated reagents are summarized. **(b)** Flow cytometric analysis of mitochondrial mass and mitochondrial membrane potential monitored by MitoTracker Green and TMRM staining on the indicated day or differentiation monitored by CD138 and IgG1 expression after 4 days of culture with LPS + IL-4 in the presence or absence of the indicated reagents are summarized. **(c)** Flow cytometric analysis of mitochondrial mass and cellular ROS monitored by MitoTracker Green and CellROX staining on the indicated day or differentiation monitored by CD138 and IgG1 expression after 4 days of culture with LPS + IL-4 in the presence or absence of the indicated reagents are summarized. Data are shown as mean ± s.e.m. #$p > 0.05$ (not significant), **$p < 0.05$, ***$p < 0.005$. (two-tailed Student's *t*-test)

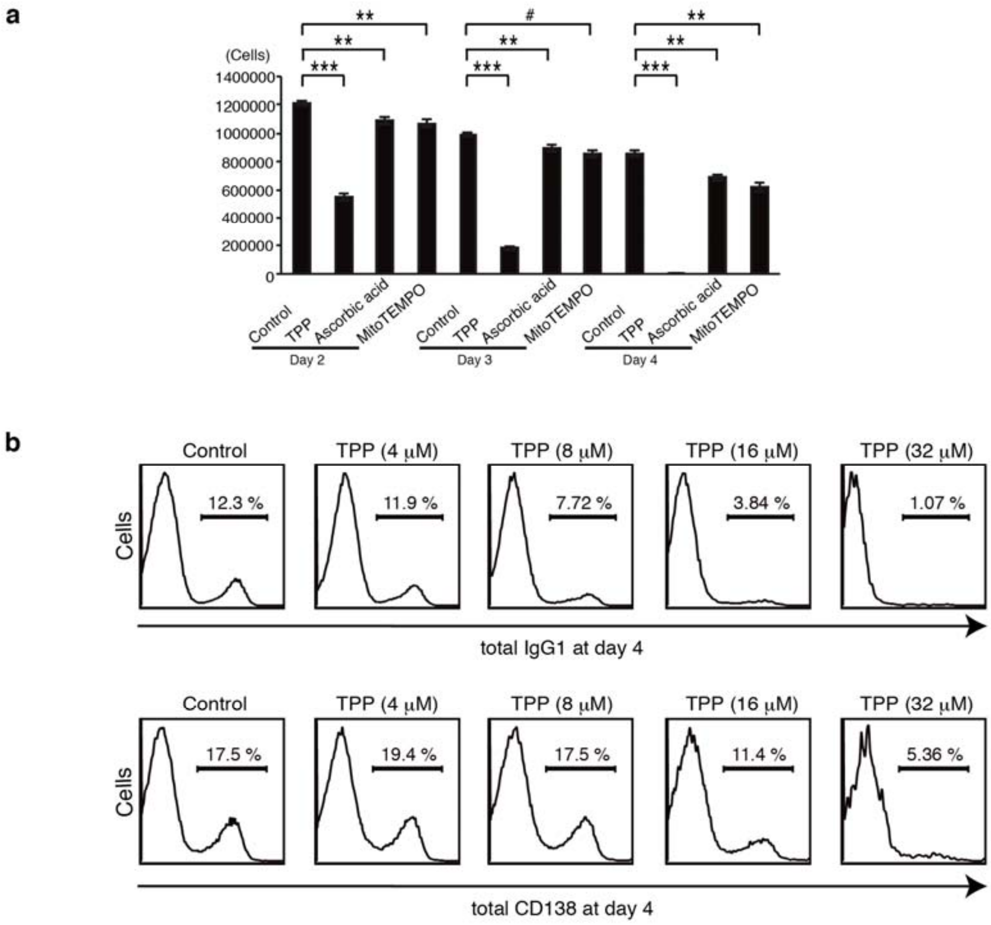

Supplementary Figure 10 Effect of TPP in activated B cells

(**a**) Cell numbers of cultured splenic B cells are plotted. (**b**) Flow cytometric analysis of the differentiation status monitored by CD138 and IgG1 expression after 4 days of culture with LPS + IL-4 in the presence or absence of indicated amounts of TPP. Data are shown as mean ± s.e.m. #$p > 0.05$ (not significant), **$p < 0.05$, ***$p < 0.005$. (two-tailed Student's *t*-test)

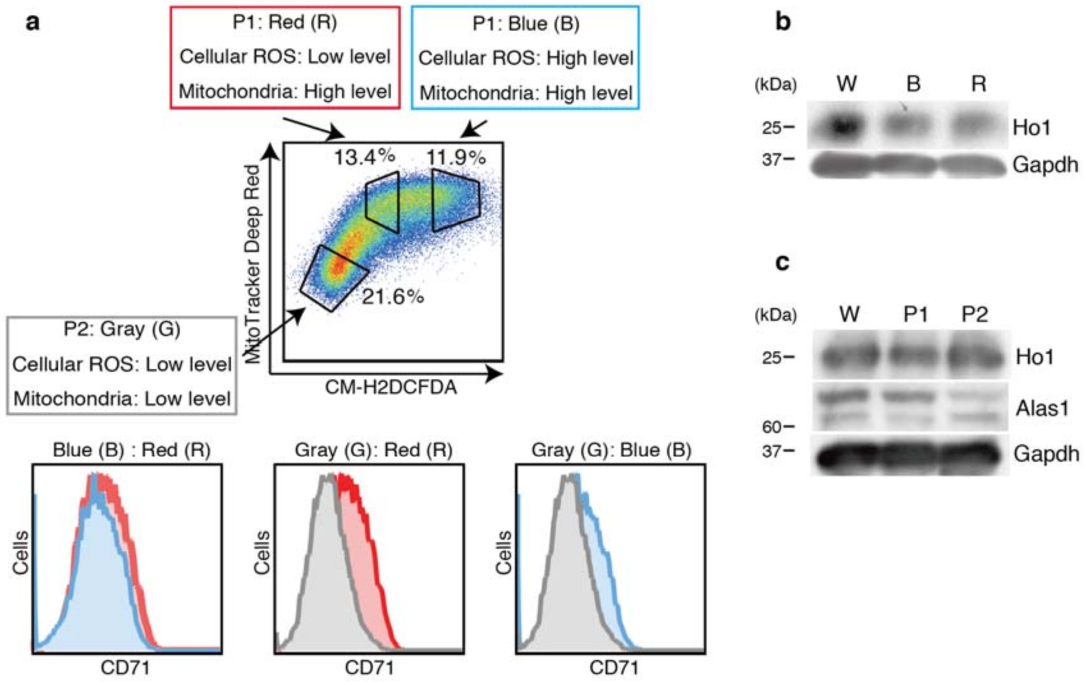

Supplementary Figure 11 CD71, HO-1, and ALAS expression in ROS^{high}, ROS^{low}, and P2 cells

(a) Flow cytometric analysis of CD71 expression in ROS^{high}, ROS^{low}, and P2 cells. **(b)** Immunoblot analysis of HO-1 in ROS^{high} and ROS^{low} cells. W: whole cell lysate, B: ROS^{high} cell lysate, R: ROS^{low} cell lysate. **(c)** Immunoblot analysis of HO-1 and ALAS in P1 and P2 cells. Data shown are representative of three independent experiments. CD71 expression in P2 cells is also shown.

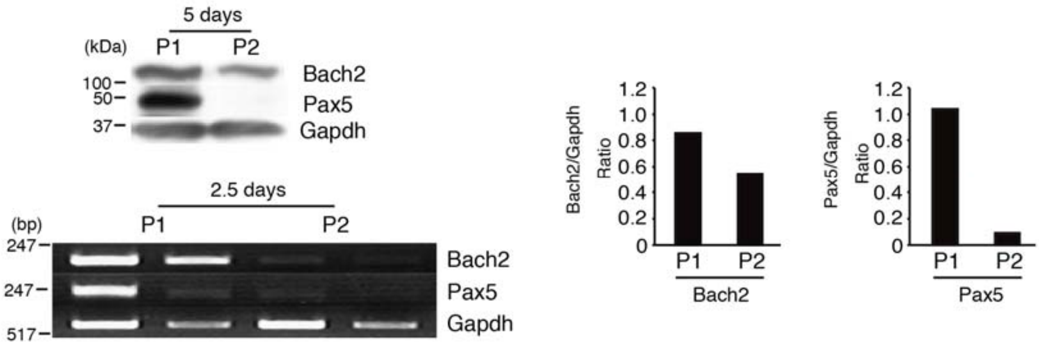

Supplementary Figure 12 Pax5 and Bach2 expressions in P1 and P2 cells after culturing 5 days

Western blotting analysis of sorted P1 and P2 cells after 5 days of culturing (left upper). Quantitative analysis of Bach2 and Pax5 proteins are plotted (right). Data shown are representative of two independent experiments. RT-PCR analysis of sorted P1 and P2 cells after 2.5 days of culturing (left lower). Five-fold serial dilutions of cDNAs were amplified for the indicated transcript. Data shown are representative of three independent experiments.

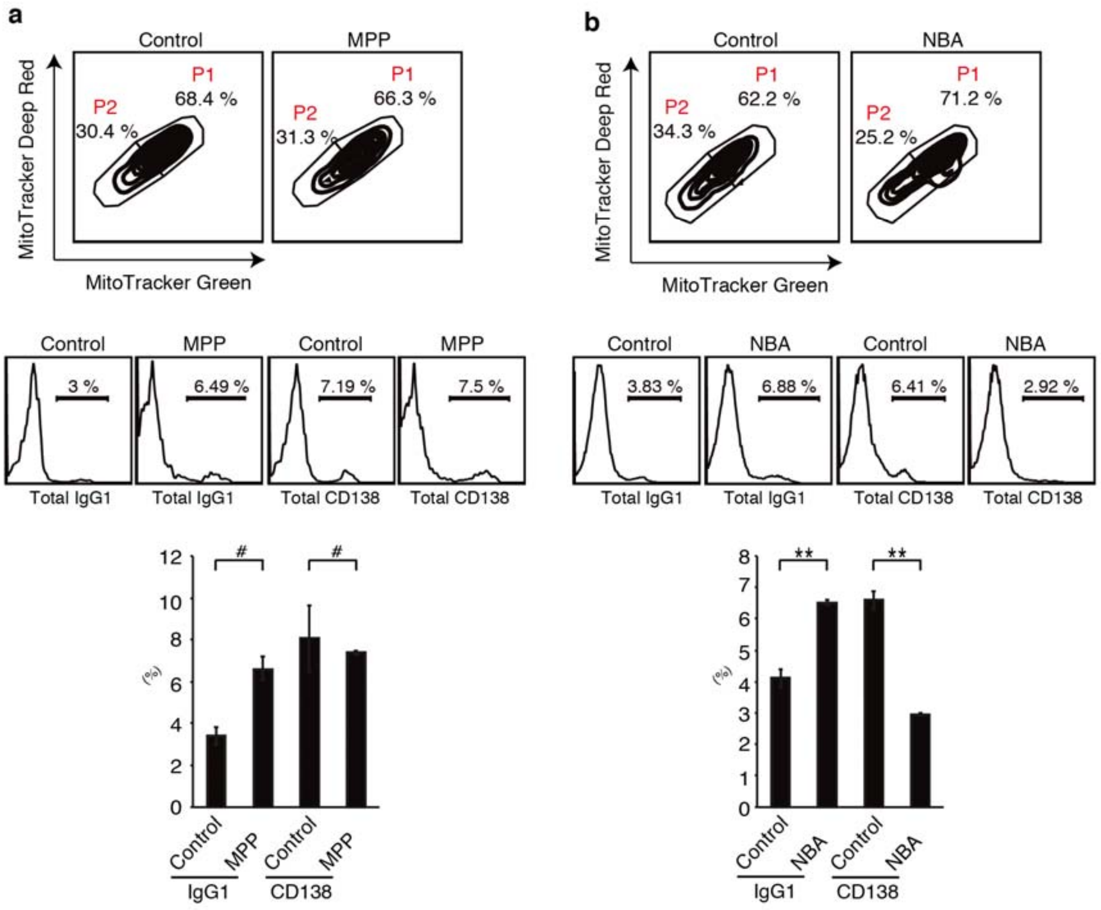

Supplementary Figure 13 Effects of ferrochelatase inhibitor and respiratory complex inhibitor on activated B cell fates *in vivo*.

(a) Flow cytometric analysis of mitochondrial status (upper) of B220$^+$NP$^+$ cells from the spleen 3 days after immunization with NP-Ficoll in the presence or absence of MPP (ferrochelatase inhibitor) and differentiation status (middle). These data are summarized in the lower panel. **(b)** Flow cytometric analysis of the mitochondrial status (upper) of B220$^+$NP$^+$ cells from the spleen 3 days after immunization with NP-Ficoll in the presence or absence of NBA (inhibitor for complexes I and III) and the differentiation status (middle). These data were summarized in the lower panel. Data shown are representative of three independent experiments. Data are shown as mean ± s.e.m. #p > 0.05 (not significant), **p < 0.05. (two-tailed Student's t-test)

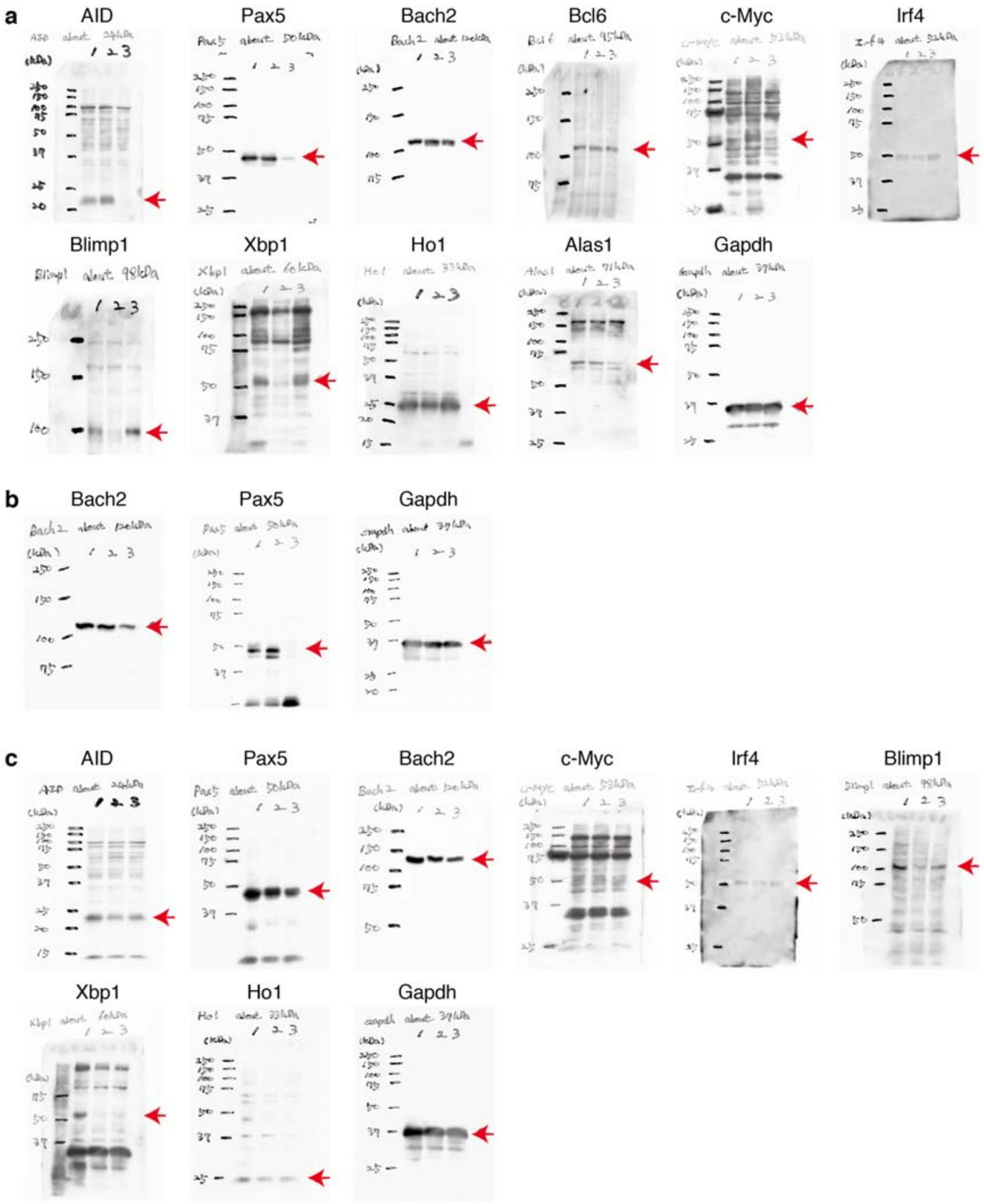
a
AID
Pax5
Bach2
Bcl6
c-Myc
Irf4
Blimp1
Xbp1
Ho1
Alas1
Gapdh
b
Bach2
Pax5
Gapdh
c
AID
Pax5
Bach2
c-Myc
Irf4
Blimp1
Xbp1
Ho1
Gapdh

Supplementary Figure 14 Full size images of all western blots

(a) Full size images of western blotting data shown in Figure 3e. **(b)** Full size images of western blotting data shown in Supplementary Figure 12 (left upper). **(c)** Full size images of western blotting data shown in Figure 6d. Red arrows indicate each expected proteins.
